# Comparative efficacy of exercise modalities for cardiopulmonary function in hemodialysis patients: A systematic review and network meta-analysis

**DOI:** 10.3389/fpubh.2022.1040704

**Published:** 2022-12-01

**Authors:** Wanli Zang, Mingqing Fang, He He, Liang Mu, Xiaoqin Zheng, Heng Shu, Nan Ge, Su Wang

**Affiliations:** ^1^Postgraduate School, University of Harbin Sport, Harbin, China; ^2^Xiangya Hospital, Central South University, Changsha, China; ^3^Winter Olympic Academy, University of Harbin Sport, Harbin, China; ^4^Postdoctoral Research Station, University of Harbin Sport, Harbin, China; ^5^School of Physical Education, South China Normal University, Guangzhou, China; ^6^Department of Sports Science, University of Harbin Sport, Harbin, China

**Keywords:** chronic kidney failure, renal dialysis, exercise, cardiopulmonary function, Bayesian network meta-analysis

## Abstract

**Background:**

To provide reliable evidence to exercise rehabilitation therapists and clinicians, we compared and analyzed the effects of different exercise modalities on cardiopulmonary function in hemodialysis patients using Bayesian network meta-analysis.

**Methods:**

PubMed, OVID, Web of Science, Cochrane Library, Embase, Scopus, CINAHL, SPORT Discus, SinoMed, CNKI, Wanfang, and VIP were searched from inception to July 20, 2022. We included randomized controlled trials comparing 12 exercise modalities to improve cardiorespiratory fitness in hemodialysis patients. All statistical analysis was performed using STATA and R.

**Result:**

A total of 82 randomized controlled trials involving 4146 maintenance hemodialysis patients were included in this study. The pair-wise meta-analysis showed that all exercise modalities had a positive effect on all indicators of cardiorespiratory capacity. The network meta-analysis demonstrated that Blood flow restriction training (BFRT), Cycle exercise (CE), Inspiratory muscle training (IMT), Combined aerobic and resistance training (CT), and Aerobic training (AT) were significantly better than usual care for 6-min walkability; Medium intensity continuous training (MICT), CT, CE, and AT were considerably better than usual care for VO_2_Peak; body and mind training (MBT) and CT significantly improved SBP compared to usual care; and only MBT was significantly better than usual care for DBP. Both the two-dimensional plot and the radar plot demonstrated that CT had the best combined-effect on each index of cardiorespiratory fitness. Subgroup and sensitivity analyses demonstrated the robustness of the results. The evidence was mainly “low” to “very low” for this network meta-analysis.

**Conclusion:**

There is no one exercise that can achieve the best effect on all of the outcomes. The benefits of MBT in decreasing arterial blood pressure are unsurpassed by other exercise methods. The intervention effect of the CT is better and more stable. Electrical muscle stimulation training (MEST) can be employed in individuals who do not wish to exercise actively but may cause an increase in blood pressure. On the basis of the characteristics of different exercise types, guidelines developers, clinicians, and patients may employ them appropriately.

**Systematic review registration:**

https://www.crd.york.ac.uk/PROSPERO/#recordDetails.

## Introduction

Chronic kidney disease (CKD) is a highly prevalent disease with high morbidity and mortality rates worldwide (1). According to the second iteration of the Global Kidney Health Atlas survey, the global median prevalence of renal replacement therapy is 759 per million population (PMP). It has become a significant public health issue with widespread concern. Maintenance hemodialysis (MHD) is the primary renal replacement therapy for individuals with end-stage renal disease. Although hemodialysis is a widespread and well-established treatment to prolong patient survival, the reduction of aerobic capacity, imbalance of the body (2), muscle mass (3), and exercise endurance lead to a lack of physical activity in patients, resulting in decreased quality of life and mobility (4, 5). Evidence suggests that peak oxygen uptake (VO_2_Peak) during cardiopulmonary exercise testing decreases significantly in patients with kidney disease as the disease progresses and that the causes of impaired cardiopulmonary function are diverse and include anemia (6, 7), neuropathy (8), and cardiac and vascular dysfunction (9). Therefore, the objective of hemodialysis has shifted from maintaining the basic survival and prolonging the lives of patients to improving the quality of life and promoting patients' return to society (10–12).

Exercise plays an increasingly important role as a promising non-pharmacological treatment to improve cardiopulmonary function in hemodialysis patients, and existing studies shows that physical activity improve cardiovascular and physical function as well as quality of life in people with MHD, with additional benefits including improved mood, enhanced appetite, and nutritional intake, leading to a virtuous cycle (13). The American Kidney Disease Foundation recommends that exercise training should be a cornerstone in the management of complications and treatment modalities for patients receiving HD. The guidelines (14) recommend assigning an appropriate exercise prescription to patients with MHD to assist them in increasing their mobility. However, there is no reliable evidence to provide an accurate and effective exercise program for patients with MHD.

When prescribing exercise for hemodialysis patients, it is essential for clinical professionals to consider the type of exercise. Numerous studies have shown that various forms of exercise have distinct movement structures (12 exercise modalities are described in detail in Supplementary Appendix B). Resistance training, which requires overcoming resistance at a constant rate, is effective in enhancing muscle strength and explosive power (15) as well as improving muscle mass (16), protein synthesis (17), and neuromuscular adaptations (15), in both young and old individuals; aerobic exercise, which requires continuous and repeated activity at a certain intensity for an extended duration, increases the ability of skeletal muscle to create energy through oxidative metabolism and improves cardiovascular function by inducing central and peripheral adaptations (18, 19); combined training requires the incorporation of aerobic and resistance exercise into the training program, which is an effective way to counteract the aging-induced decline in cardiorespiratory endurance and muscular strength (20); physical and mental activity is gradually gaining scholarly attention along with some other novel forms of exercise, each of which has its own characteristics depending on their different training forms. Nevertheless, the most effective exercise therapy in improving cardiorespiratory function in this population remains unclear, as no studies have simultaneously compared different types of exercise interventions. As a result, healthcare professionals have experienced difficulties in developing the most effective exercise interventions for treating patients. To overcome the limitations of traditional meta-analysis, this systematic review was conducted using a Bayesian network meta-analysis of randomized controlled trials to evaluate the effectiveness of different exercise modalities on cardiorespiratory fitness in patients with MHD.

## Methods

The systematic review was reported in accordance with the Preferred Reporting Items for Systematic Reviews and Meta-Analyses for Network Meta-Analyses (PRISMA-NMA).

### Registration

This study was registered with PROSPERO, number: CRD42022308176.

### Search strategy

An electronic search of databases including PubMed, OVID, Web of Science, Cochrane Library, Embase, Scopus, CINAHL, SPORT Discus, SinoMed, CNKI, Wanfang Data, and VIP was performed from inception to June 12, 2022. We adopt personalized retrieval based on the characteristics of each database. Details of search terms and search strategies are supplied in Supplementary Appendix A. ClinicalTrials.gov and the Chinese Clinical Trial Registry were also searched for potential unpublished trials. Furthermore, the reference lists of all included research and associated meta-analyses were combed for additional relevant studies. We repeated the searches before formal analysis to supplement our study. When it was not available from the literature, we contacted the authors to obtain the required data.

### Study selection

We first eliminated duplicates using EndNote 20.2.1 software (Thomson Research Soft, USA). Then, two researchers independently screened titles and abstracts to identify all potentially relevant studies. Studies that met the inclusion criteria were independently identified and assessed by the same two authors. Any disagreement was decided by a third reviewer. Detailed inclusion criteria are listed below: (1) The experimental design must be a randomized controlled trial (RCT); (2) All participants must be maintenance hemodialysis patients (≥18 years old); (3) The intervention includes any one of 12 exercises (see Supplementary Appendix B for details and abbreviations of the exercise classification); (4) At least one of the following outcomes must be reported in studies: 6-min walkability, peak oxygen uptake, systolic blood pressure (SBP), and diastolic blood pressure; and (5) literatures published in any language. The reasons for excluding studies are as follows: (1) the subjects of the study were minors; (2) The patients were not on maintenance hemodialysis; (3) Exercise is compared with other interventions with the exception of usual care (e.g., physical therapy); and (4) The data were not presented in the required format, and the authors did not respond to our request.

### Data extraction

Two researchers performed data extraction using a document information extraction form created by Microsoft Excel (Microsoft Corporation, Redmond, Washington, version 16.0). The extracted information included first author, year of publication, trial sample size, baseline information of hemodialysis patients, method of random sequence generation, intervention of trial and control groups, exercise prescription, measured outcomes, adverse effects, and mean and standard deviation (SD) of outcomes. Literature screening and data extraction were conducted independently by two reviewers, with a third reviewer engaging in the discussion and decision-making when there was disagreement.

### Risk of bias assessment

Two researchers used the Risk of Bias Assessment Tool for Randomized Trials (RoB2.0) (21) recommended by the Cochrane Collaboration. The five domains were as follows: “Randomization process,” “Deviations from intended interventions,” “Mising outcome data,” “Measurement of the outcome,” and “Selection of reported result.” The risk of bias can be divided into three levels: “low risk of bias,” “some concern,” and “high risk of bias.” Disagreements were resolved by consensus or by a third reviewer.

### Statistical synthesis and analysis

The pair-wise meta-analysis was conducted through R (4.1.3), using the meta package (22), and the random effects model was selected to account for heterogeneity among studies. We did not conduct a meta-analysis of interventions that had only one or two studies (23). We determined heterogeneity by examining forest plots, and the *I*^2^ statistic. For *I*^2^ >25%, we choose the random effects model; for *I*^2^ <25%, we choose the fixed effects model.

Non-informative priori Bayesian Network meta-analysis (NMA) was performed in R using the “BUGSnet” (24) and “gemtc” (25) packages with specific parameters: 100,000 simulation iterations and 10,000 adaptation iterations. Markov chain Monte Carlo simulations were used for each outcome, with a thinning interval of 10, indicating that one sample was collected every 10 iterations. Convergence was assessed graphically using trajectory and density plots, and the Brooks-Gelman-Rubin statistics were calculated to quantify the convergence. For the network meta-analysis, we fitted a Bayesian random effects model in R using the “BUGSnet” package and assumed a common heterogeneity across treatment comparisons. Data is presented as mean differences and 95% confidence intervals to allow rehabilitation therapists to assess the effectiveness of exercise. Usual care was chosen as a control group to compare with other exercise methods in the forest plot. *I*^2^ test was applied to evaluate heterogeneity. The node splitting method (26) and posterior distribution plot (27) were used to check the local and global consistency, respectively. The surface under the cumulative ranking area (SUCRA) (27) was calculated using the probability ranking table provided by the “BUGSnet” package to rank the efficacy of the interventions. The SUCRA ranges from 0 to 1, with higher values indicating more effective improvements in cardiorespiratory fitness. Specifically, among the four outcomes, higher values indicate increased 6-min walk capacity, increased peak oxygen uptake, and decreased systolic and diastolic blood pressures. We used meta-regression to analyze the association between improvements in cardiorespiratory fitness and covariates (age and gender); Subgroup analyses were conducted to explore whether there were differences in efficacy of exercise in dialysis or non-dialysis settings; and sensitivity analysis (excluding infrequently used exercise methods based on four outcomes to test the stability of the results) was combined with two-dimensional plots and radar plots to identify the more desirable exercise modality. Heat plots were generated to summarize the effects of all exercise modalities on cardiopulmonary function. Finally, comparison-adjusted funnel plots were generated using STATA 15.0 (28) software (Stata Corporation, College Station, TX, USA) to test for publication bias in this study.

### CINeMA evaluation

We assessed the certainty of the evidence contributing to the network estimation of the outcomes using the confidence in network meta-analysis (CINeMA) framework. The CINeMA (29) evaluation consists of six evaluable items: within-study bias, reporting bias, indirectness, imprecision, heterogeneity, and incoherence. There are four levels of evidence: high, medium, low, and very low, and the grade of RCT is high before evaluation. The level of evidence is decreased by one level or two levels for each nonconformity of the above six components.

## Results

**Figure 1:**
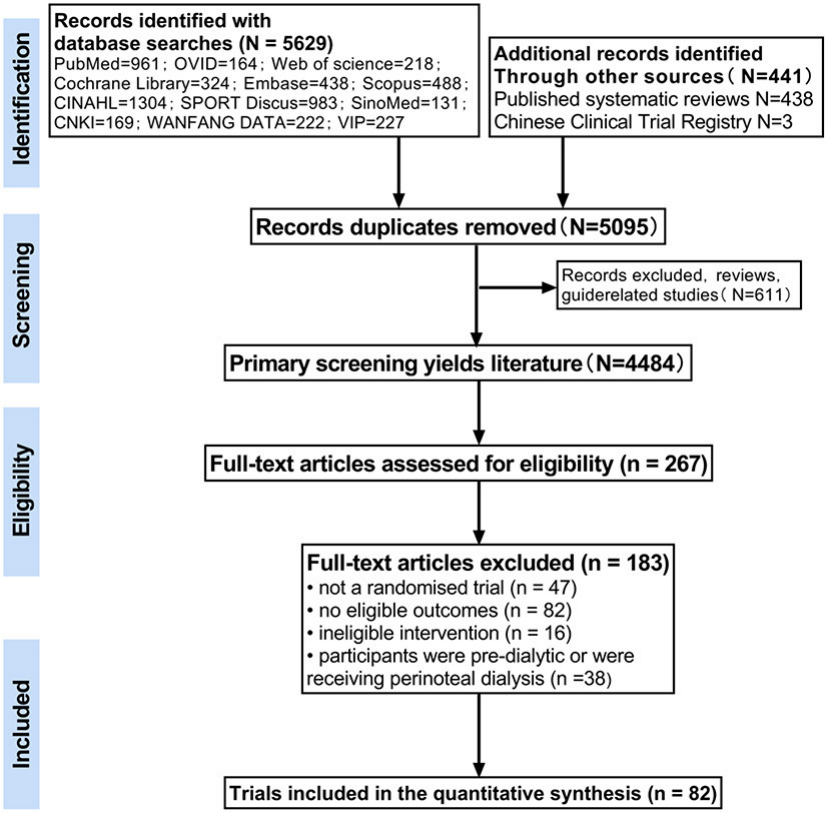
PRISMA flow diagram chart for systematic review and network meta-analysis.

The initial search identified 6,071 studies, of which 5,629 were obtained by searching 12 databases through the established search strategy, 438 were discovered by scanning published systematic reviews, and four ongoing studies were obtained from the Clinical Trial Registry. Of these, 976 were excluded because of duplication. Based on the inclusion and exclusion criteria, 82 publications were finally obtained and included in the meta-analysis (the flow chart of study selection is shown in Figure 1). One unpublished study from the Clinical Trial Registry was obtained by contacting the author. The trials were conducted in both developed and developing countries and involved 4,147 patients with kidney disease requiring hemodialysis (literature characteristics are shown in Supplementary Appendix C). The majority of included studies were of poor methodological quality, with 37% reporting adequate randomization and only 7.4% at low overall risk of bias. The risk of bias assessment for each study is shown in Supplementary Appendix D(a), and the summary risk of bias assessment is shown in Supplementary Appendix D(b).

### Pair-wise meta-analysis

#### Six-minute walking capacity

In a meta-analysis of 52 studies, the researchers discovered that exercise significantly improved 6-min walking ability compared with usual care (MD = 37.93 m, *p* < 0.001, 95% CI 29.27–46.6, *I*^2^ = 53.6%). CT, CE, and AT achieved an extremely significant effect (*p* < 0.001); RT showed a significant effect (0.001 < *p* < 0.05); IMT and MEST did not achieve a significant effect [Supplementary Appendix E–B(a)].

##### VO_2_Peak

A meta-analysis of 21 studies showed that exercise increased VO_2_Peak significantly compared with usual care (MD = 3.61 ml/kg/min, *p* < 0.001, 95% CI 2.76–4.46, *I*^2^ = 42.8%). CT, CE, and AT all achieved an extremely significant effect [*p* < 0.001; Supplementary Appendix E–B(b)].

##### SBP

A meta-analysis of 26 studies revealed that exercise had a highly significant reduction in SBP compared with usual care (MD = −6.1 mmHg, *p* < 0.001, 95% CI −8.47 to −3.74, *I*^2^ = 49.4%). MBT had a highly significant effect (*p* < 0.001); CT and AT has a significant effect (0.001 < *p* < 0.05); CE did not achieve a significant effect [Supplementary Appendix E–B(c)].

##### DBP

A meta-analysis of 33 studies indicated that exercise significantly reduced DBP compared with usual care (MD = −2.68 mmHg, *p* = 0.0128, 95% CI −4.52 to −0.83, *I*^2^ = 69.4%). MBT achieved a highly significant effect (*p* < 0.001); CT achieved a significant effect; CE and AT were better than usual care, but the difference was not statistically significant [Supplementary Appendix E–B(d)].

##### Network meta-analysis

The posterior mean deviance contributions of the four outcomes indicated the contributions to the deviance were very similar and close to 1, for both the consistency and inconsistency models. The node splitting method showed no statistically significant differences between direct and indirect comparisons for VO_2_Peak, SBP, and DBP, except for three nodes in 6-min walking ability. The leverage plots showed that the DIC of the consistency model was the smallest. Therefore, the consistency model was chosen (Supplementary Appendix F). Trace and density plots as well as Brooks-Gelman-Rubin (PSRF = 1) indicated good convergence of the model.

**Figure 2:**
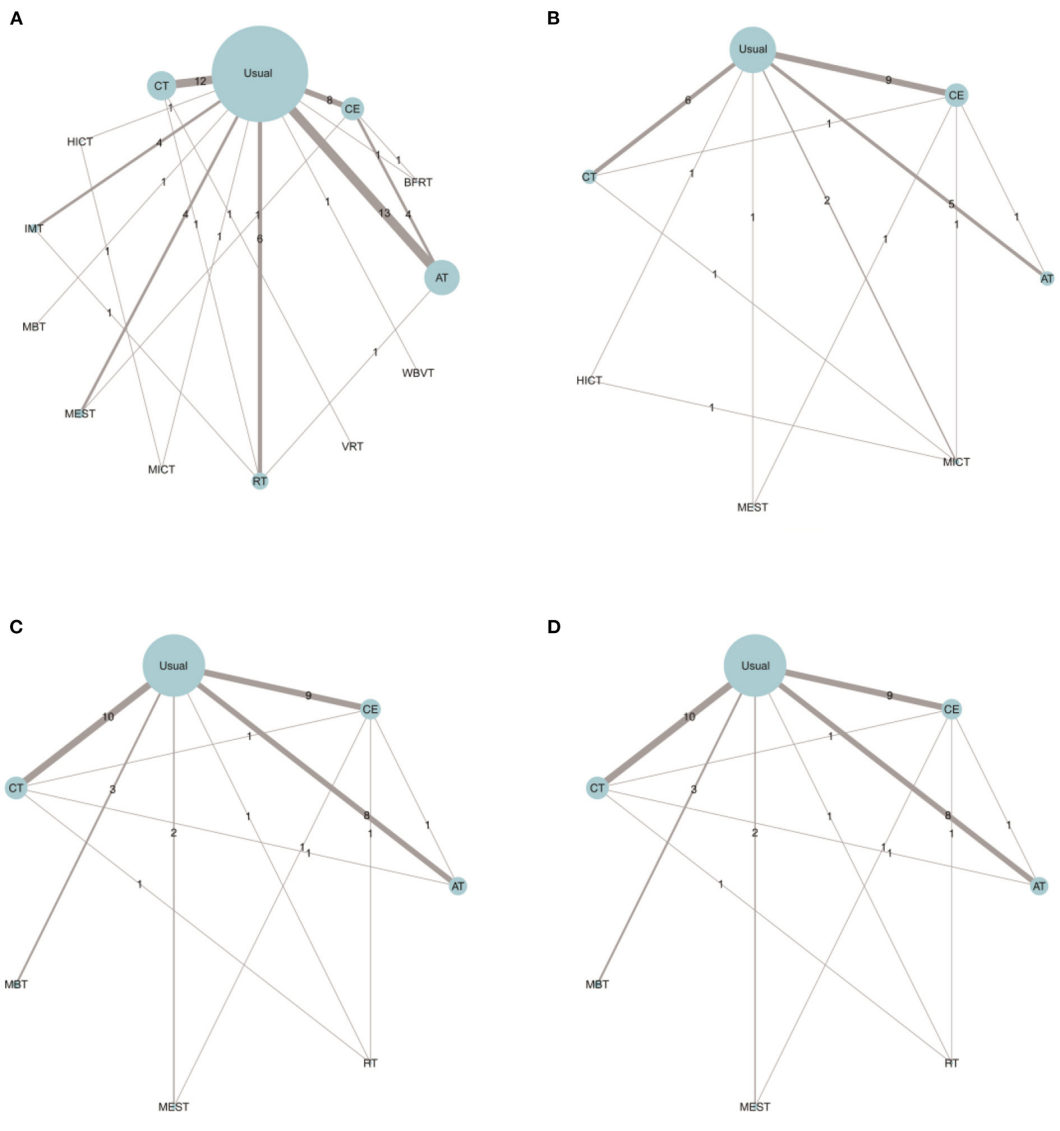
Comparing the effects of different exercise modalities 6WMT **(A)**, VO_2_Peak **(B)**, SBP **(C)**, and DBP **(D)** in hemodialysis patients. AT, aerobic training; Usual, usual care; CT, combined training; RT, resistance training; CE, cycle dynamometers; MEST, electrical muscle stimulation; IMT, inspiratory muscle training; BFRT, blood flow restriction exercise; HICT, high-intensity circuit training; MICT, medium intensity continuous training; VRT, virtual reality training; MBT, body and mind training.

**Figure 3:**
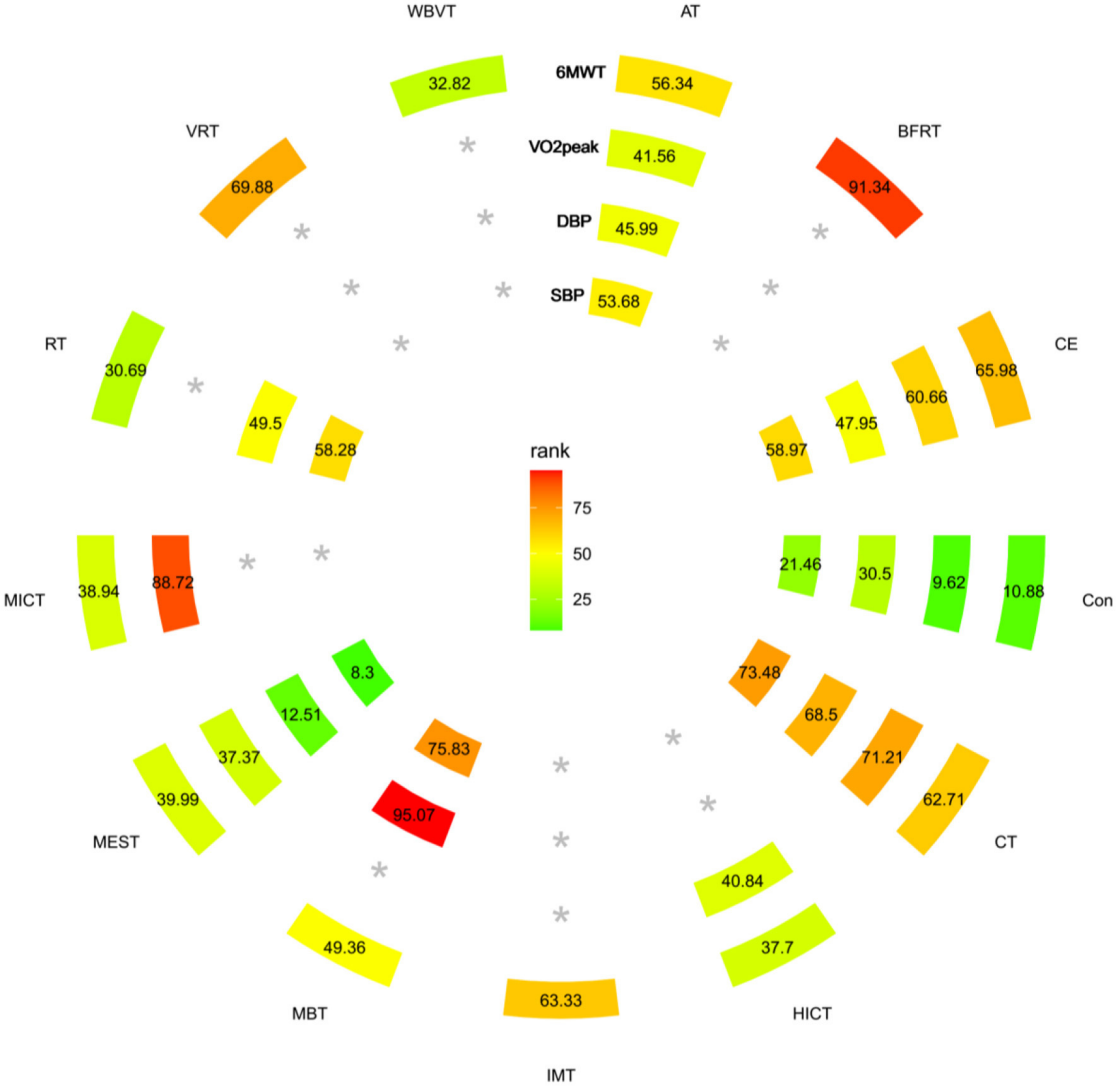
Heat map ranked according to the degree of relevant change in 6WMT, VO_2_Peak, SBP, and DBP parameters. The numbers reflect the *P*-score, ranked continuously from 0 to 1. A higher SUCRA indicates a more significant increase in 6WMT, VO_2_Peak. In the case of blood pressure parameters, a higher SUCRA suggests a more pronounced effect on lowering blood pressure. ^*^The exercise mode corresponding to the outcome was not included in the literature. ^*^Treatment without data on the outcome within the circle.

**Figure 4:**
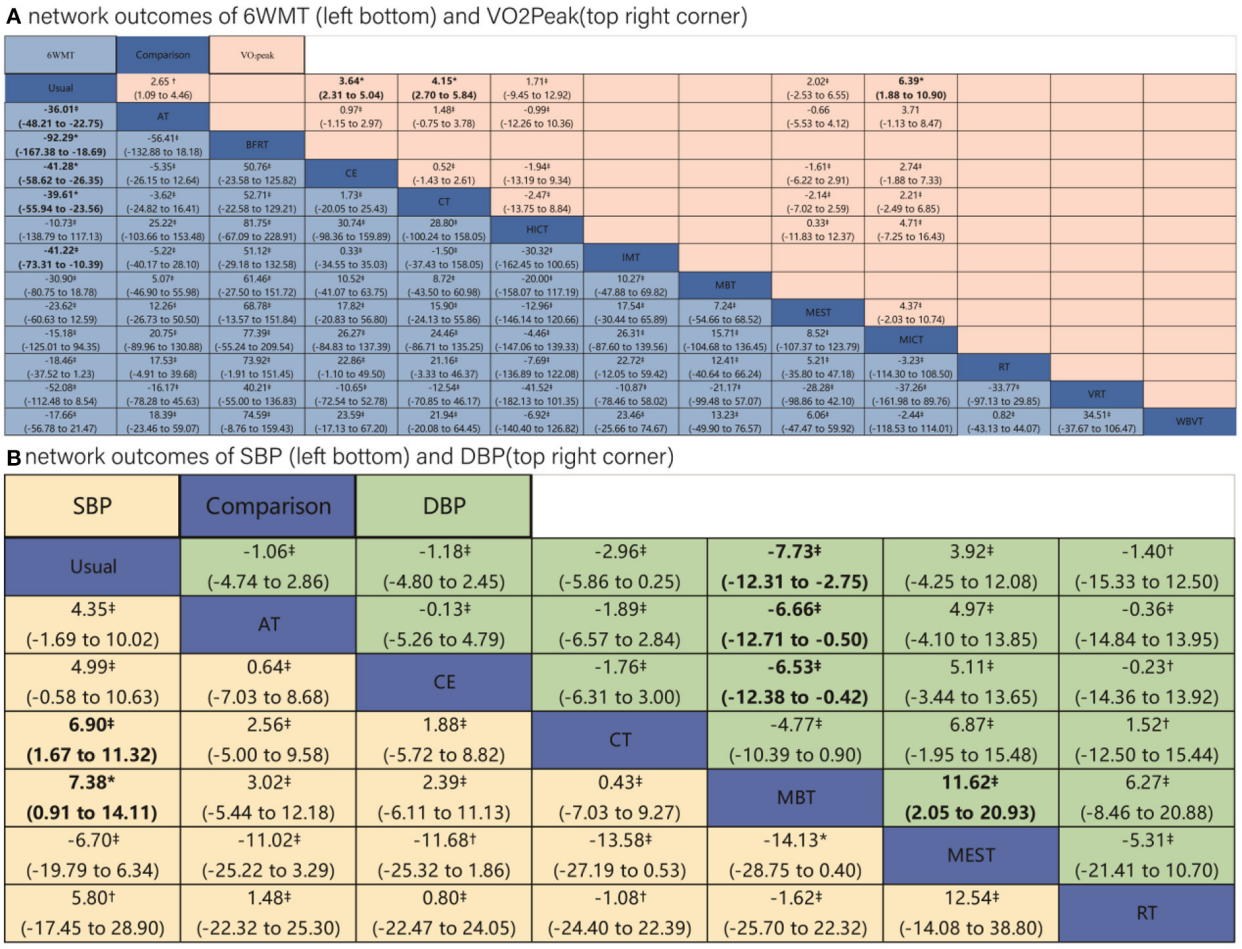
Results of network meta-analysis of four outcomes. Comparisons should be read from left to right. **(A)** Outcomes of 6 WMT and VO_2_Peak. **(B)** Outcomes of SBP and DBP. Outcomes are located at the intersection between the column-defining treatment and the row-defining treatment. Data are in MD (95% CIs). For 6MWT and VO_2_Peak, MDs above 0 favor the column-defining treatment. For SBP and DBP, MDs below 0 favor the column-defining treatment. The certainty of the evidence [according to confidence in network meta-analysis (CINeMA)] was incorporated in this figure as footnotes. ^*^Moderate quality of evidence. ^†^Low quality of evidence. ^‡^Very low quality of evidence. The significant results in league tables have been bolded. AT, aerobic training; Usual, usual care; CT, combined training; RT, resistance training; CE, cycle dynamometers; MEST, electrical muscle stimulation; IMT, inspiratory muscle training; BFRT, blood flow restriction exercise; HICT, high-intensity circuit training; MICT, medium intensity continuous training; VRT, virtual reality training; MBT, body and mind training.

The trial network plots for each outcome were drawn to show the interventions in the network meta-analysis (Figure 2). The width of the lines is proportional to the number of trials for each pair of interventions, and the size of each node is proportional to the number of participants assigned to that intervention. We also summarized the SUCRA for each intervention through heat maps (Figure 3). Comparisons of the efficacy of any two interventions are shown in Figure 4.

#### Six-minute walkability

A total of 52 RCTs were included (30–81), of which five three-arm trials and 47 two-arm trials, involving 12 interventions (CT, CE, HICT, BFRT, IMT, AT, MBT, WBVT, MEST, VRT, MICT, and RT). The network meta-analysis showed that AT, BFRT, CE, CT, and IMT were significantly superior to usual care. However, there was no significant difference between them (*p* > 0.05). HICT, MBT, WBVT, MEST, VRT, MICT, and RT showed no significant difference compared with usual care. The SUCRA of 6-min walking ability in descending order were BFRT, VRT, CE, IMT, CT, AT, MBT, MEST, MICT, HICT, RT, and WBVT.

##### VO_2_Peak

A total of 21 RCTs were included (34, 43, 55, 72, 78, 82–97), of which one four-arm trial, two three-arm trials, and 18 two-arm trials, involving six interventions (AT, CE, CT, HICT, MEST, and MICT). The network meta-analysis showed that AT, MICT, CE, and CT were significantly better than the usual care, but there was no significant difference between each other (*p* > 0.05). HICT and MEST were not significantly different from usual care. The SUCRA of VO_2_Peak in descending order were MICT, CT, CE, AT, HICT, and MEST.

##### SBP

A total of 26 RCTs (31, 38, 46, 60, 63, 73, 77, 82, 83, 87, 88, 90, 93, 98–110) were included, of which one four-arm experiment, three three-arm experiments, and 24 two-arm experiments, involving six interventions (AT, CE, CT, MBT, MEST, and RT). The network meta-analysis revealed that MBT and CT2 intervention measures were significantly better than usual care, but there was no significant difference between each other (*p* > 0.05). There was no significant difference in AT, CE, RT, and MEST compared with usual care. The SUCRA of SBP in descending order were MBT, CT, CE, RT, AT, and MEST.

##### DBP

A total of 26 RCTs (31, 38, 46, 60, 63, 73, 77, 82, 83, 87, 88, 90, 93, 98–110) were included, of which one four-arm experiment, 3 three-arm experiments, and 24 two-arm experiments involving six interventions (AT, CE, CT, MBT, MEST, and RT). Network meta-analysis showed that MBT was significantly better than usual care, and there was no significant difference in AT, CE, CT, MEST, and RT compared with usual care. The SUCRA of DBP in descending order were MBT, CT, CE, RT, AT, and MEST.

##### Comparison of pair-wise meta-analysis and network meta-analysis

The comparison of pair-wise meta-analysis and network meta-analysis revealed that the results were generally consistent (Supplementary Appendix F). Only a small difference was found in the 6-min walkability results of IMT: pair-wise comparison of IMT vs. control [*I*^2^ = 71.7%; 51.94 (−3.61; 107.49), *p* = 0.067] vs. network comparison of IMT vs. control [41.22 (10.39–73.31), *p* < 0.05].

In the two-dimensional plots based on SUCRA values, CT, CE, and AT are distributed around a 45-degree diagonal line, proving that these interventions have a balanced effect on the promotion of cardiopulmonary function. And CT had the best intervention effect (furthest from zero), followed by CE and AT (shown in Supplementary Appendix G). Subgroup analyses explored the effects of several settings on the effectiveness of exercise. The CE and AT performed in a non-dialysis setting were superior to CT in improving 6-min walking ability.AT is more effective than CE for DBP in the dialysis environment. In the remaining outcomes, the ranking of the effects of the three most commonly used interventions remained consistent with CT being the most effective, followed by CE and AT (Supplementary Appendix H). Sensitivity analyses of the most commonly used exercise methods showed no substantial change in outcomes (Supplementary Appendix I).

The results of meta-regression revealed that, with the exception of the VO_2_Peak, other outcomes were not substantially influenced by covariates (Supplementary Appendix J). We showed the CINeMA assessment in the league table (Figure 4). The comparisons of Usual care with CE, CT, and BFRT achieved moderate quality of evidence for the outcome of 6WMT; the comparison of Usual care with CE, CT, and MEST achieved moderate quality of evidence for the outcome of VO_2_Peak; and the comparison between Usual care and MBT and between MBT and MEST achieved moderate quality of evidence for the outcome of SBP. The quality of evidence was “low” to “very low” for comparisons between other interventions for the four outcomes (details were shown in Supplementary Appendix K).

### Publication bias

The horizontal coordinate of comparison-adjusted funnel plots indicates effect sizes, and the vertical coordinate represents the inverse of the standard error. All comparisons were symmetrically distributed on the whole and concentrated at the top of the funnel, indicating a lower risk of publication bias. The small number of studies was scattered at the bottom, possibly related to the small sample size (Supplementary Appendix K).

## Discussion

Our network meta-analysis assessed the relative efficacy of different exercise interventions on 6WMT, VO_2_Peak, SBP, and DBP in hemodialysis patients. The purpose of this study is to explore the relative effectiveness of different exercise modalities on cardiorespiratory fitness and thus enrich the understanding of exercise rehabilitation therapy in the field of complementary alternative medicine. The meta-analysis was based on studies from 82 studies with a total of 4,146 individuals. Fifty-three RCTs tested the efficacy of 12 exercise interventions on improving 6-min walk capacity in patients with MHD; 21 RCTs evaluated the effect of six exercise modalities on VO_2_Peak; 26 RCTs assessed the impact of six exercise modalities on arterial blood pressure. Three interventions, AT, CE, and CT, are more prevalent in clinical practice, and Some more novel exercise modalities, such as MEST, IMT, and WBVT, have emerged in clinical trials of interventions for patients with MHD. To our knowledge, this is the first network meta-analysis to compare the efficacy of over a dozen exercise methods in hemodialysis patients. The results demonstrated that BFRT and MICT were the most effective methods in improving 6-min walking capacity and VO_2_Peak, respectively. MBT was the best in improving diastolic and systolic blood pressure. CT, CE, and AT improved all outcomes to varying degrees. The results of MEST were the worst for VO_2_Peak, SBP and DBP, and only this type of exercise can raise blood pressure in hemodialysis patients, which is harmful to their physical health.

Currently, the awareness that sports rehabilitation training has a positive impact on functional level and physical health has been widely recognized in the rehabilitation medicine. For instance, exercise increases the level of prostacyclin in patients and strengthens plasma fibrinolysis and antithrombosis, thereby relaxing blood vessels, reducing blood viscosity, improving vascular compliance, decreasing peripheral resistance, and lowering diastolic blood pressure; Sports can reduce the excitability of sympathetic nerves, decrease the release of catecholamine in plasma or lower catecholamine sensitivity, and relax vascular smooth muscle accordingly, consequently reducing systolic blood pressure (111–114); Exercise can increase the release of endorphins and 5-HTP, meanwhile, reduce plasma epinephrine and aldosterone levels, thus attenuating vasoconstriction and water and sodium retention of the renin-angiotensin-aldosterone system to lower blood pressure. Sports increase adaptation to the cardiopulmonary and neuromuscular systems, enhance oxygen delivery to the mitochondria and regulate muscle metabolism more stringently (115–117). There are various forms of exercise rehabilitation, and the effects of different types of rehabilitation exercise are also different.

Of the published studies evaluating health status of MHD patients, 6WMT is the most commonly used outcome, whereas VO_2_Peak has been reported less frequently probably due to the challenge of measuring this outcome in most elderly patients. Although 6WMT which was proven to be relatively and absolutely accurate in maintenance hemodialysis patients is clinically employed as an indicator of exercise tolerance and provides comparable prognostic information to VO_2_Peak, we still tend to use the VO_2_Peak indicator. The reason for using this is that VO_2_Peak, which is obtained in the cardiopulmonary exercise test (CPET), represents a person's functional aerobic capacity. VO_2_Peak has become the gold standard for cardiopulmonary health and is inversely associated with cardiovascular risk and all-cause mortality (118). Additionally, we evaluated blood pressure, an indirect indicator of cardiopulmonary function. The two apparent changes in hemodialysis patients were arteriosclerosis and atherosclerosis, characterized by loss of arterial buffering and catheter function, and intimal thickness, resulting in increased blood pressure and decreased cardiopulmonary function (119).

The results of the direct meta-analysis demonstrated that exercise has an active effect on the health improvement of dialysis patients but not all exercise significantly improved physical function. This study yielded several new meta-analyses, such as the effect of MBT on blood pressure in hemodialysis patients, and the results showed statistically significant difference. Scapini et al. (120) reported that AT had no significant influence on SBP or DBP in hemodialysis patients. Through the inclusion of more studies, this study shows that AT has a significant effect on systolic blood pressure but not on diastolic blood pressure in hemodialysis patients. As a commonly used intervention, RT is rarely applied to improve cardiopulmonary function due to inconsistent intervention effects. Three common interventions (CE, AT, and CT) showed stable efficacy, with significant benefits for all measures except DBP, and CT significantly lowered DBP.

The results of pair-wise meta-analysis and network meta-analysis were generally consistent. In the 6-min walkability indicator, comparisons of IMT vs. control between pair-wise and network meta-analysis showed small differences. This may be due to the inclusion of less studies, large heterogeneity between studies, and the wide confidence intervals. The network meta-analysis combined direct and indirect comparisons with a larger sample size of evidence, hence narrowing the confidence interval for the effect size. In conjunction with the information in the CINeMA evaluation, we believe that the network results of the IMT vs. usual care converge more closely to the actual effect. The results of this network meta-analysis differ from the previous ones conducted by Scapini et al. There are multiple reasons. Firstly, this network meta-analysis aimed to systematically evaluate the efficacy of different exercise methods on cardiopulmonary function. Instead, Scapini et al. focused on the effects of exercise on health-related indicators, which was fragmentary. Secondly, our study involved 82 experiments (One clinical experiment registry provided experimental data), and only 31 experiments were included by Scapini et al. (120). Thirdly, we classified the exercises as accurately as possible (twelve in all), rather than simply lumping very different exercises into one category, and previous studies included only three types of exercise. Previous studies used both VO_2_Max and VO_2_Peak for oxygen uptake; however, we only considered VO_2_Peak because there is evidence that there are discrepancies in the assessment of these two indicators, which will undoubtedly affect the accuracy of the results. Consistent with the previous review, the effect of exercise depends on the type and design of exercise, and the effect of CT is superior to AT and RT among several Indicators of evaluation. This is the first study to examine whether exercise on dialysis or on non-dialysis affects treatment outcomes, with performing a subgroup analysis. Among the eight evaluations of the four indicators, the probability of CT being better than AT and CE is very high, and the probability of CE being better than AT is also very high. To further explore the comprehensive influence of exercise on cardiopulmonary function in patients with hemodialysis, we also conducted a comprehensive evaluation using two-dimensional plots, with one group consisting of VO_2_Peak and 6WMT and the other group consisting of DBP and SBP. CT, CE, and AT are distributed along the slope of 45 degrees, far from the zero, indicating that they have positive effects in different outcomes, and CT is better than CE and AT. Finally, we used the radar plots to observe the combined effect of three most commonly used exercise modes on the four indicators. The area enclosed by the curve of CT was the largest, which indicated the best effect, followed by CE, and at was the least. In order to check robustness of the results, we performed a sensitivity analysis, which increased the effect of CT on 6WMT, indicating that CT has a stable benefit in treating cardiopulmonary function in MHD patients. It is noteworthy that MEST, although capable of improving 6WMT and VO_2_Peak, has a side effect of increasing blood pressure, which is consistent with the conclusion of Stefan et al. (121). Therefore, clinical use of MEST should be targeted based on its characteristics. MBT shows a better performance than CT for the blood pressure reduction, but the comparison of related studies on other indicators is scarce, thus its efficacy cannot be comprehensively evaluated. The majority of the evidence in this study was low to extremely low, and the efficacy of the less studied exercise methods was only only for the reference of clinicians or rehabilitation therapists.

To help the exercise prescription of hemodialysis patients, we scanned the studies that included CT and CE exercise prescriptions. When using CE, researchers more often require patients to exercise by Borg's RPE scale at 12–16, namely, moderate to vigorous intensity. Interestingly, although CE is included in AT, it is possible to increase resistance to increase strength, so enhancing both aerobic capacity and muscle strength. Although the evidence indicates that the intervention effect of CE is slightly lower than CT, we guess this may be related to the proportion of oxygen and resistance. As we all know, the energy supply of all exercise is provided by three energy supply systems (ATP-CP system, glycolysis system, and aerobic system) in different proportions, depending on the nature and characteristics of exercise. Obviously, CE needs to maintain a certain period of movement, and the resistance strength is not large enough. In CT-related exercise prescriptions, aerobic and resistance exercises are typically completed in the same training session, referred internationally to as “Concurrent Training” or “Concurrent Strength and Endurance Training.” Wilson et al. (122) contend that concurrent training is a training method for gaining strength, muscle hypertrophy, explosive resistance training, and endurance-enhancing training in the same training stage. Davis et al. (123) think that concurrent training can maintain the strength level of the body and improve endurance and other essential physical qualities without reducing the strength level. Concurrent training benefits than traditional CT training. Given the excellent performance of concurrent training, combined with the exercise prescriptions of the included studies, we recommend that hemodialysis patients use concurrent training as a training tool during rehabilitation. The majority of hemodialysis patients are elderly, inactive, and are less able to adapt to physical loads. Therefore, a low-intensity adaptation phase needs to be arranged in the first weeks of training, with improving aerobic capacity as the primary objective. Multiple sessions (3, 4) of aerobic endurance training can be arranged each week to enhance the body's ability to adapt and recover, so as to build the foundation of physical health. As the training cycle progresses, the proportion of aerobic training decreases. Concurrent training is increased to maintain aerobic capacity while increasing strength development, thus improving overall physical fitness and promoting healthy growth. While considering the specific design of concurrent training, the rehabilitation therapist should also prescribe exercise based on the patient's personal preferences, which will significantly improve compliance with long-term exercise.

## Strengths/limitations

Several methodological strengths exist in this systematic review, focusing on the review of currently popular exercises for hemodialysis patients (CT, CE, and AT), with a preliminary exploration of some novel interventions. And more than a dozen databases were searched with a view to a comprehensive and systematic literature search. This is the first systematic review of the effects of exercise on hemodialysis patients in a dialysis setting or non-dialysis setting. For the first time in this field, meta-regression analysis and CINeMA evidence have been used, which greatly enhanced the credibility of the results. Although the network meta-analysis compensated for the lack of pair-wise meta-analysis, there were some limitations to the inclusion of studies due to the clinical characteristics of exercise interventions for hemodialysis patients. This study did not consider the exercise environment, and there may be differences in the effect of exercise between exercise at home and community centers. The general physical condition of the patients, various comorbidities, and medications were not considered, which may affect the effect of exercise. In selecting indicators to measure cardiopulmonary function, the applicable indicators were not selected, which may cause some bias in evaluating the efficacy of exercise. The quality of majority of the literature has certain risks, such as blinded and allocation concealment, and the level of evidence is generally “low” to “very low.” Although 82 randomized controlled trials were Included, Involving 4,146 patients and dozens of exercise modalities, with the exception of three commonly used exercise modalities, there were few studies on other exercises, and the ranking of overall efficacy may be biased. Most studies a small sample size due to conditions of clinical trials, which may lead to bias; it is unclear whether the effects of these interventions change over time because the studies were conducted over a short period of time.

## Conclusion

There is no single exercise modality that is optimal for all indicators. MBT is significantly more effective than other forms of exercise for lowering blood pressure; CT is a superior and more stable intervention MEST can be applied to patients who are reluctant to exercise actively but may cause an increase in blood pressure. The evaluation of the effectiveness of interventions contributes evidence-based practice, and guideline makers, physicians, and patients can refer to the characteristics of different exercise modalities for precise application. Due to some limitations of existing clinical studies and evidence, future studies should focus on larger sample sizes, longer follow-ups, and developing training programs (intensity, frequency, duration).

## Data availability statement

The original contributions presented in the study are included in the article/Supplementary material, further inquiries can be directed to the corresponding author.

## Author contributions

WZ, LM, XZ, HS, and SW: research idea and study design. WZ, XZ, HS, NG, LM, and HH: data acquisition. WZ, MF, LM, and SW: data analysis/interpretation. WZ, MF, LM, HH, and SW: statistical analysis. LM, XZ, SW, HH, and MF: supervision or mentorship. All authors contributed important intellectual content during manuscript drafting or revision and accept accountability for the overall work by ensuring that questions pertaining to the accuracy or integrity of any portion of the work are appropriately investigated and resolved.

## Conflict of interest

The authors declare that the research was conducted in the absence of any commercial or financial relationships that could be construed as a potential conflict of interest.

## Publisher's note

All claims expressed in this article are solely those of the authors and do not necessarily represent those of their affiliated organizations, or those of the publisher, the editors and the reviewers. Any product that may be evaluated in this article, or claim that may be made by its manufacturer, is not guaranteed or endorsed by the publisher.
