# Supplementary material for: Comparative efficacy of exercise modalities for cardiopulmonary function in hemodialysis patients: A systematic review and network meta-analysis
Source: Front Public Health. 2022 Dec 1;10:1040704. doi: 10.3389/fpubh.2022.1040704 (PMC9751492; doi:10.3389/fpubh.2022.1040704)
Supplement: Supplementary file 1 [file Data_Sheet_1.pdf]

Appendix

Appendix A. Search strategy.（p1-6）
Appendix B. Details and abbreviations of exercise classification.（p7）
Appendix C. Characteristics of the studies included in the Systematic review.（p8-30）
Appendix D. Risk of bias assessment.（p31-32）
Appendix E. Comparisons with usual care.（p33-37）
Appendix F. Model selection.（p38-41）
Appendix G. Comprehensive assessment.（p42）
Appendix H. Sub-group analysis.（p43）
Appendix I. Sensitivity analysis.（p44）
Appendix J. Regression analysis.（p45-49）
Appendix K. Confidence in network meta analysis (CINeMA)（p50-61）
Appendix L. Publication bias.(p62-63)

Appendix A. Search strategy.

PubMed
#1 (intradialytic[tiab] OR intra-dialytic[tiab] OR hemodialysis[tiab] OR haemodialysis[tiab] OR renal dialysis[MeSH] OR extracorporeal dialysis OR extracorporeal dialyses OR dialysis[MeSH] OR dialyses OR HD) AND (6MWT OR six-minute walk test OR 6-minute walk test OR 6-min walk test OR VO_2peak OR VO_2 OR blood pressure OR systolic blood pressure OR diastolic blood pressure)
#2 exercise[MeSH] OR exercises OR resistance exercise OR resistance training[MeSH] OR strength training OR aerobic exercise OR aerobic exercises OR exercise aerobic OR aerobic training OR aerobic versus strength OR strength versus aerobic OR physical fitness[MeSH] OR physical training OR exercise therapy[MeSH] OR combined training OR aerobic plus strength training OR aerobic plus resistance exercise OR aerobic plus resistance training OR concurrent training OR concurrent exercise OR electrostimulation OR electro-stimulation OR inspiratory muscle training OR Body-Mind Exercise OR tai chi or yoga OR qigong OR Baduanjin OR Traditional Chinese Exercise
#3 randomized controlled trial[pt] OR controlled clinical trial[pt] OR clinical trial[pt] OR randomized controlled trials[mh] OR random allocation[mh] OR double-blind method[mh] OR singleblind method[mh] OR clinical trial[pt] OR clinical trials[mh] OR ("clinical trial"[tw]) OR ((singl*[tw] OR doubl*[tw] OR trebl*[tw] OR tripl*[tw]) AND (mask*[tw] OR blind*[tw])) OR ("latin square"[tw]) OR placebos[mh] OR placebo*[tw] OR random*[tw] OR research design[mh:noexp] OR follow-up studies[mh] OR prospective studies[mh] OR cross-over studies[mh] OR control*[tw] OR prospectiv*[tw] OR volunteer*[tw] NOT (animal[mh] NOT human[mh])
#1 AND #2 AND #3

Cochrane Library
#1MeSH descriptor: [Renal Dialysis] explode all trees<br>#2MeSH descriptor: [Dialysis] explode all trees<br>#3intradialytic or hemodialysis or haemodialysis or extracorporeal dialysis or extracorporeal dialyses or dialyses or HD<br>#4#1 or #2 or #3<br>#5MeSH descriptor: [Exercise] explode all trees<br>#6MeSH descriptor: [Resistance Training] explode all trees<br>#7MeSH descriptor: [Physical Fitness] explode all trees<br>#8MeSH descriptor: [Exercise Therapy] explode all trees<br>#9exercises or resistance exercise or strength training or aerobic exercise or aerobic exercises or exercise aerobic or aerobic training or physical training or combined training or aerobic plus strength training or aerobic plus resistance exercise or aerobic plus resistance training or concurrent training or concurrent exercise or aerobic versus strength or strength versus aerobic or inspiratory muscle training or IMT or electrostimulation or electro-stimulation or Body-Mind Exercise or tai chi or yoga or qigong or Baduanjin or Traditional Chinese Exercise<br>#10 #5 or #6 or #7 or #8 or #9<br>#11 6MWT or six minute walk test or 6 minute walk test or 6 min walk test or VO_2peak or VO_2 or blood pressure or systolic blood pressure or diastolic blood pressure<br>#12 #4 and #10 and #11 in Trials

Embase
#1 intradialytic OR haemodialysis OR renal dialysis OR extracorporeal dialysis OR extracorporeal dialyses OR dialyses OR HD OR 'hemodialysis'/exp OR 'dialysis'/exp<br>#2 exercises OR resistance exercise OR strength training OR aerobic exercises OR exercise aerobic OR aerobic training OR physical fitness OR physical training OR exercise therapy OR combined training OR aerobic plus strength training OR aerobic plus resistance exercise OR aerobic plus resistance training OR concurrent training OR concurrent exercise OR Body-Mind Exercise OR tai chi OR yoga OR qigong OR Baduanjin OR Traditional Chinese Exercise OR 'exercise'/exp OR 'training'/exp OR 'resistance training'/exp OR 'aerobic exercise'/exp OR electrostimulation OR electrostimulation OR inspiratory muscle training<br>#3 random$ OR doubl$ adj blind$ OR singl$ adj blind$ OR assign$ OR allocat$ OR 'randomized controlled trial'/exp<br>#1 AND #2 AND #3

Scopus
["Physical Therapy Modalities" OR "Modalities, Physical Therapy" OR "Modality, Physical Therapy" OR "Physical Therapy Modality" OR "Physiotherapy (Techniques)" OR "Physiotherapies (Techniques)" OR "Physical Therapy Techniques" OR "Physical Therapy Technique" OR "Techniques, Physical Therapy" OR "Group Physiotherapy" OR "Group Physiotherapies" OR "Physiotherapies, Group" OR "Physiotherapy, Group" OR "Physical Therapy" OR "Physical Therapies" OR "Therapy, Physical" OR "Neurological Physiotherapy" OR "Physiotherapy, Neurological" OR "Neurophysiotherapy" OR "exercise" OR "Exercises" OR "Physical Activity" OR "Activities, Physical" OR "Activity, Physical" OR "Physical Activities" OR "Exercise, Physical" OR "Exercises, Physical" OR "Physical Exercise" OR "Physical Exercises" OR "Acute Exercise" OR "Acute Exercises" OR "Exercise, Acute" OR "Exercises, Acute" OR "Exercise, Isometric" OR "Exercises, Isometric" OR "Isometric Exercises" OR "Isometric Exercise" OR "Exercise, Aerobic" OR "Aerobic Exercise" OR "Aerobic Exercises" OR "Exercises, Aerobic" OR "Exercise Training" OR "Exercise Trainings" OR "Training, Exercise" OR "Trainings, Exercise"] AND ["Renal Dialysis" OR "Dialyses, Renal" OR "Renal Dialyses" OR "Dialysis, Renal" OR "Hemodialysis" OR "Hemodialyses"] AND ["randomized controlled trial " OR "randomized" OR "placebo" OR "random"] AND (LIMIT-TO (SUBJAREA , "NURS") OR LIMIT-TO (SUBJAREA , "HEAL")) AND (EXCLUDE (SUBJAREA , "AGRI") OR EXCLUDE (SUBJAREA , "ARTS") OR EXCLUDE (SUBJAREA , "BIOC")) AND (EXCLUDE (SUBJAREA , "BUSI") OR EXCLUDE (SUBJAREA , "COMP") OR EXCLUDE (SUBJAREA , "EART") OR EXCLUDE (SUBJAREA , "ENGI") OR EXCLUDE (SUBJAREA , "IMMU") OR EXCLUDE (SUBJAREA , "MATE") OR EXCLUDE (SUBJAREA , "MATH") OR EXCLUDE (SUBJAREA , "MEDI") OR EXCLUDE (SUBJAREA , "NEUR")) AND (EXCLUDE (SUBJAREA , "PHAR") OR EXCLUDE (SUBJAREA , "PSYC") OR EXCLUDE (SUBJAREA , "SOCI"))

SPORTDiscus
#1 (MH "dialysis" OR "hemodialysis") OR intradialytic OR haemodialysis OR renal dialysis OR extracorporeal dialysis OR extracorporeal dialyses OR dialyses OR HD<br>#2 (MH "exercise" OR "resistance training" OR "physical fitness") OR exercises OR resistance exercise OR strength training OR aerobic exercise OR aerobic exercises OR exercise aerobic OR aerobic training OR aerobic versus strength OR strength versus aerobic OR physical training OR exercise therapy OR combined training OR aerobic plus strength training OR aerobic plus resistance exercise OR aerobic plus resistance training OR concurrent training OR concurrent exercise OR Body-Mind Exercise OR tai chi or yoga OR qigong OR Baduanjin OR Traditional Chinese Exercise<br>#3 ((MH "randomized controlled trials") OR controlled clinical trial OR random*) NOT (PT review)<br>#1 AND #2 AND #3

CINAHL
#1 (MH "dialysis" OR "hemodialysis") OR intradialytic OR haemodialysis OR renal dialysis OR extracorporeal dialysis OR extracorporeal dialyses OR dialyses OR HD<br>#2 (MH "exercise" OR "resistance training" OR "physical fitness") OR exercises OR resistance exercise OR strength training OR aerobic exercise OR aerobic exercises OR exercise aerobic OR aerobic training OR aerobic versus strength OR strength versus aerobic OR physical training OR exercise therapy OR combined training OR aerobic plus strength training OR aerobic plus resistance exercise OR aerobic plus resistance training OR concurrent training OR concurrent exercise OR electrostimulation OR electro-stimulation OR inspiratory muscle training OR Body-Mind Exercise OR tai chi or yoga OR qigong OR Baduanjin OR Traditional Chinese Exercise<br>#3 ((MH "randomized controlled trials") OR controlled clinical trial OR random*) NOT (PT review)<br>#1 AND #2 AND #3

Web of science
#1 ((((((TS=(dialysis)) OR TS=(hemodialysis)) OR TS=(intradialytic)) OR TS=(renal dialysis)) OR TS=(extracorporeal dialysis)) OR TS=(extracorporeal dialyses)) OR TS=(HD)
#2(((((((((((((((((((((((TS=(exercises)) OR TS=(resistance exercise)) OR TS=(strength training)) OR TS=(aerobic exercise)) OR TS=(aerobic exercises)) OR TS=(exercise aerobic)) OR TS=(aerobic training)) OR TS=(aerobic versus strength)) OR TS=(strength versus aerobic)) OR TS=(physical training)) OR TS=(exercise therapy)) OR TS=(combined training)) OR TS=(aerobic plus strength training)) OR TS=(aerobic plus resistance exercise)) OR TS=(aerobic plus resistance training)) OR TS=(concurrent training)) OR TS=(concurrent exercise)) OR TS=(electrostimulation)) OR TS=(electro-stimulation)) OR TS=(inspiratory muscle training)) OR TS=(Body-Mind Exercise)) OR TS=(taichi)) OR TS=(yoga)) OR TS=(qigong)) OR TS=(Baduanjin)) OR TS=(Traditional Chinese Exercise)
#3(((((((TS=(6MWT)) OR TS=(six-minute walk test)) OR TS=(6-minute walk test)) OR TS=(6-min walk test)) OR TS=(VO_2peak)) OR TS=(blood pressure)) OR TS=(systolic blood pressure)) OR TS=(diastolic blood pressure)
#4 (((TS=(randomized controlled trial)) OR TS=(controlled clinical trial)) OR TS=(clinical trial)) OR TS=(random allocation)
#1 AND #2 AND #3 AND #4

OVID
#1 Exercises OR resistance exercise OR strength training OR aerobic exercise OR aerobic exercises OR exercise aerobic OR aerobic training OR aerobic versus strength OR strength versus aerobic OR physical training OR exercise therapy OR combined training OR aerobic plus strength training OR aerobic plus resistance exercise OR aerobic plus resistance training OR concurrent training OR concurrent exercise OR electrostimulation OR electro-stimulation OR Body-Mind Exercise OR taichi OR yoga OR qigong OR Baduanjin OR Traditional Chinese Exercise
#2 renal dialysis OR extracorporeal dialysis OR extracorporeal dialyses OR HD renal dialysis<br>haemodialysis
#3 randomized controlled trial OR controlled clinical trial OR clinical trial OR random allocation
#1 AND #2 AND #3

SinoMed
#1 Exercise OR Activity OR Training OR Exercise OR Sports<br>#2 Dialysis OR Renal Dialysis OR Haemodialysis<br>#3 Oxygen uptake OR VO_2Peak OR Blood pressure OR SBP OR DBP OR Six-minute walk capacity OR 6MWD<br>#4 Randomised OR Randomised controlled OR Randomised controlled trial OR RCT

CNKI
(((((subject%='haemodialysis' or title%='haemodialysis') OR(old subject= haemodialysis + dialysis + kidney dialysis + kidney disease)) AND((subject%= 'exercise' or title%= exercise') OR(old subject= exercise + exercise + physical activity + physical activity + aerobic exercise + resistance exercise + combined exercise + activity + training + blood flow restriction exercise + Tai Chi + Qigong + Mind-Body Exercise + Yoga + Whole Body Vibration + Eight Duan Jin + Five Animal Play + Bicycle)) AND(old version of topic= xls(VO_2Peak) + Six Minute Walkability + Six Minute Walkability + Blood Pressure + Systolic Blood Pressure + Diastolic Blood Pressure)) AND((topic%=''Randomised Control' or title%=''Randomised Control') OR(l day version of topic= Randomised Control + Randomised Controlled Experiment + Randomised)))

WANFANG DATA
((Topic: Haemodialysis) or (Title or keyword: Haemodialysis or Dialysis or Renal Dialysis or Kidney Disease)) and ((Topic: Exercise) (Title or keyword: Exercise or Exercise or Physical Activity or Physical Activity or Aerobic Exercise or Resistance Exercise or Combined Exercise or Activity or Training or Blood Flow Restriction Exercise or Tai Chi or Qigong or Body and Mind Exercise or Yoga or Whole Body Vibration or Eight Duan Jin or Five Animal Play or Pedal Bicycle)) and ((Title or keyword: VO_2Peak or six-minute walking capacity or six-minute walking capacity or blood pressure or systolic blood pressure or diastolic blood pressure)) and ((Topic: Randomised Control) (Title or keyword: Randomised Control or Randomised Controlled Trial or Randomised))

VIP
(M=Hemodialysis or U= (Haemodialysis or Dialysis or Renal Dialysis or Renal Disease)) AND (M=Exercise or U= (Exercise or Exercise or Physical Activity or Physical Activity or Aerobic Exercise or Resistance Exercise or Blood Flow Restriction or Tai Chi or Qigong or Body-Mind Exercise or Yoga or Whole Body Vibration or Eight Duan Jin or Five Animal Play or Foot Bicycle)) AND (U= (VO_2Peak or six-minute walking capacity or six-minute walking capacity or blood pressure or systolic blood pressure or diastolic blood pressure)) AND (M=randomised control or U=(randomised control or randomised controlled trial or randomised))

Appendix B. Details and abbreviations of motion pattern classification.

Definitions of exercise training interventions and non-exercise training controls		
Type	abbreviations	define
aerobic training	AT	Exercises training such as walking, cycling (This study does not include pedal bikes with adjustable resistance) and jogging in any land-based mode that is designed to improve the efficiency and capacity of the cardiorespiratory system
Blood flow restriction exercise	BFRT	Compression device restricts blood flow to distal muscles to achieve high intensity strength training through less intense muscle stimulation
Body and mind training	MBT	Physical activity while concentrating to ensure mental focus and awareness, including a range of different forms of exercise and practice
combined training	CT	Exercise program includes aerobic and resistance exercises
Cycle Dynamometers	CE	Cyclic dynamometer with adjustable resistance
Electrical muscle stimulation	MEST	A technique that applies a low-frequency current of 20-50Hz through electrodes to stimulate specific muscle groups to make them twitch or contract and then achieve "functional" repair.
High-intensity circuit training	HICT	High-intensity circuit training, often unassisted, lasting several minutes
Inspiratory muscle training	IMT	Promote the function of respiratory muscles by adjusting breathing patterns
Medium intensity continuous training	MICT	Moderate intensity circuit training, often unassisted, for longer durations.
resistance training	RT	Exercise training designed to improve the strength, power, endurance and size of skeletal muscles
Virtual Reality Training	VRT	The use of computers and their peripherals to simulate a three-dimensional virtual motion environment, which allows users to create a sense of immersion
Whole Body Vibration Training	WBVT	Through the physical machinery repeatedly vertical up and down vibration vibration generated by the frequency and amplitude combined with the principle of resonance effectively on the human body neuromuscular firing feedback

Appendix C. Characteristics of the studies included in the review.

Study	County	Croup (n)	Age (years)	Gender		Interventions			
				female	male	Type	Period	Prescription	Intensity level
Marieke2005	Netherlands	53	52±15	34	19	CT	ALL	12 weeks； 2 ~ 3/week; The first two months (5 to 10 minutes warm up + 20 minutes exercise + 5 to 10 cool down); Last month (lasting20 to 30 minutes)	RPE12
		43	58±16	30	13				
Dobsak2011	Czech Republic	11	58.2±7.2			CE	ID	10 weeks； 3/week; The first 5 weeks (5 minutes warm up + 20 minutes riding + 5 minutes cool down)after5 weeks ， increased to 2x20minutes	60%W_{peak}
		11	64.5±8.1			MEST		10 weeks； 3/week; Lasts 60 minutes ("on-off" mode stimulus (20-s stimulation, 20-s rest), pulse width 200 ms, rise and fall time 1 s, and amplitude 60 mA.)	Frequency 10 Hz
		10	60.1±8.2						
Stephanie2016	USA	8	66.9	8	0	CE	ID	12 weeks； 3/week; 5 minutes warm up + lasts 15 minutes (2.5 minutes per week increase) + 5 minutes cool down	RPE12 ~ 14
		7	57.9	6	1	RT		12 weeks； 3/week; Ankle weights were used for knee extension, knee flexion and	RPE12 ~ 14

Study	County	Croup (n)	Age (years)	Gender		Interventions			
				female	male	Type	Period	Prescription	Intensity level
								hip flexion. A Thera-Band was used for hip abduction	
		8	60.3	3	5	CT		12 weeks; 3/week; Resistance + bicycle	RPE12 ~ 14
		8	49.3	7	1				
Tadashi2018	Japan	13	66.2±12.8	12	1	MEST	ID	8weeks ; 3/week; Each duty cycle included a 5-s stimulation period with a 2-s pause for a period of 20 min using a monophasic, exponential climbing pulse (10).	30.6 to 104.0 mA; frequency 20 Hz
		13	65.1±8.1	12	1				
Molsted2004	Denmark	11	59 (25-58)	---	---	CT	ND	5 months; 2/week; Lasts 1 hour (10 minutes warm up + 20 to 30 minutes strength and aerobic combined training+ dynamometer exercise of different intensities 15 ~ 20 minutes + 5 to 10cool down)	RPE17
		8	48 (23-58)	---	---				
Vince2002	Canada	20	55±16	10	10	CT	ID	12 weeks; 3/week; 20 minutes of aerobic exercise + 10 minutes of strength training for the hamstrings and quadriceps group in a dialysis chair (gradually increasing	"somewhat strong" At approximately 50 rpm

Study	County	Croup (n)	Age (years)	Gender		Interventions			
				female	male	Type	Period	Prescription	Intensity level
								the number of times)	
		17	54±14	13	4			12 weeks； 3/week; 30 minutes of non-progressive, resistance-free. low-intensity, lower and free upper limb range of motion	
Pomidori2016	Italy	22	63±15	15	7	CE	ND	6 months; 10-minute walk twice a day on non-dialysis days	70% baseline speed to 120%.
		20	69±10	13	7	AT			
Yongyao2014	China	32	45（37-48）	5	27	CE	ID	12 weeks； 3/week; Lasts20 minutes (5 minutes warm up + 10 to 15 minutes recumbent bicycle).	RPE12-16
		33	44（41-50）	5	28			12 weeks； 3/week; Stretch for 1 0-15 min	
Petraki2008	Greece	22	50.0±13.2	15	7	CT	ID	7 months; 3/week; 60 min of cycling and 30 min strengthening and flexibility exercises； RT consisted of sets of reps of isotonic	RPE13
		21	50.5±14.4	17	4				
FlaviaBaria2014	Finland	10	52.1±11.4			AT	ALL	12 weeks； 3/week; Lasts for 40 minutes	40%-

Study	County	Croup (n)	Age (years)	Gender		Interventions			
				female	male	Type	Period	Prescription	Intensity level
								(5 minutes warm up + formal practice 30 minutes + 5 minutes cool down)	60%VO_2Max
		9	53.4±9.6						
Kouidi2009	Greece	24	46.3±11.2	14	10	CE	ID	3/week; 60 min ergometer cycling and 20 min strengthening exercises; RT initially consisted of2 sets of exercises for the lower limbs using elastic bands and free weights. The workload was gradually increased by increasing the number of reps (8 to 12) and the number of sets.	RPE11-13
		20	45.8±10.9	12	8				
Deligiannis1999	Greece	16	46.4±13.9	11	5	CT	ND	6 months; 3/week; Lasts for 90 minutes (10 minutes warm up + 50 intermittent aerobic + 10cool down)	60%-70%HRmax
		10	51.4±12.5	8	2	AT		6 months; 5/week; Lasts 30 minutes	60% Hrmax
		12	50.2±7.9	4	8				
Koufaki2002	UK	18	57.8±14.3	13	5	CE	ID	3 months; 3/week;6-8 minutes rides, gradually increasing the duration, at the end of the training can complete 2 20 minutes workouts or last 30-35 minutes	RPE at the 90% of VT power output

Study	County	Croup (n)	Age (years)	Gender		Interventions			
				female	male	Type	Period	Prescription	Intensity level
		15	51±18.9	11	4				
Konstantinidou2002	Greece	10	46.4±13.9	8	2	MICT	ID	6 months; 3/week; Lasts for 60 minutes (10 minutes warm up + 30 intermittent aerobic + 10cool down).	60%-70%HRmax
		10	48.3±12.1	8	2	CT	ID	6 months; 3/week; 70 min (5 min warm up + 60 min combined aerobic and impedance training + 5 min cool down).	70%HRmax
		12	51.4±12.5	4	8	CE		6 months; 5/week; 30 minutes ride + some simple flexibility and muscle stretching exercises	50%-60%HRmax
		15	50.2±7.9	8	7		ND		
Mustata2011	Canada	8	64（55,73）	---	---		ND	1 year; 2/week for the first two months; The third month begins 3/week; Lasts 5-20 minutes, gradually increasing to 60 minutes	RPE12-15
		10	72（59,79）	---	---				
Tsuyuki2003	Japan	17	40.1±11.9	9	8	AT	ND	20 weeks；2-3/week; 30 minute aerobic	50%-60%HRmax
		12	39.7±10.7	5	7				

Study	County	Croup (n)	Age (years)	Gender		Interventions			
				female	male	Type	Period	Prescription	Intensity level
Reboredo2018	Brazil	12	50.7±10.7	5	7	CE	ID	12 weeks； 3/week; 10 minutes warm up + 35 minutes pedal bike practice + 3 minutes cool down	RPE4-6
		12	42.2±13	5	7				
Kirsten2010	Australia	15	52.3±10.9	11	4	CE	ID	6 months; 2/week; Practice for at least 15 minutes each for the first two weeks; Start of week 12 and last 30 minutes each time; Each session begins on week 24 and lasts 45 minutes	RPE12-13
		15	52.1±13.6	10	5	AT		6 months; 3/week; Practice for at least 15 minutes each for the first two weeks; Each session begins on week 24 and lasts 45 minutes	RPE12-13
		16	51.3±14.4	8	8				
Cheema2007	New Zealand	24	60±15.3	17	7	RT	ID	12 weeks； 3/week; Two sets of 8 repetitive 10 exercises were performed for the main muscle groups of the upper and lower extremities	RPE15-17
		25	62.6±14.2	17	8				
Bobby2007	Australia	20	60±15.3	---	---	RT	ID	24weeks； 3/week; Two sets of 8 repetitions of10 PRT exercises were	RPE15-17

Study	County	Croup (n)	Age (years)	Gender		Interventions			
				female	male	Type	Period	Prescription	Intensity level
								performed	
		19	65±12.9	---	---				
Lucía2020	Spain	24	65.3±15.2	15	9	CE	ID	16weeks； 3/week; 10-30 minutes by bike	RPE12-15
		22	61.9±12.1	14	8	AT	ND	16weeks； 3/week; 15-30 min walk	RPE12-15
Antonio2019	Brazil	20	44.3±11.3	---	---	AT	ID	8weeks； 3/week; Lasts 50 minutes (10 minutes warm up + 30 minutes bench bike + 10 minutes cool down)	50%-70%HRmax
		19		---	---				
Gordon2018	United Kingdom	17	52.1（44.2-59.9）	14	3	CE	ID	10 weeks； 3/week; 5 minutes warm up + 50-60 minutes riding practice + 5 minutes cool down	RPE12-14
		16	51.5（42.3-60.6）	13	3	MEST		10 weeks； 3/week; 5 min warm up + 30 min stimulation, followed by 60 min + 5 min cool down with maximum tolerated intensity	Frequency 4Hz
		18	54.3（46-62.5）	11	7				

Study	County	Croup (n)	Age (years)	Gender		Interventions			
				female	male	Type	Period	Prescription	Intensity level
Fabio2016	Italy	104	63±13	64	40	AT	ID	6 months; Family walking	Low to moderate
		123	64±14	68	55				
Jociane2016	Brazil	11	59（45-72）	9	2	MEST	ID	8weeks； 3/week; Break times decrease with the progress of the protocol (starting at 50 s and decreasing by 10 s every 2 weeks), three times a week for 8 weeks, and increase conversation time (starting at 20 min, increasing by 2 min per week).	The frequency is 80 Hz, the pulse width is 400 ms, and the shrinkage time is 10 s
		10	64.5（57.5-67.75）	8	2				
Rossella2018	Italy	53	73±5	34	19	AT	ND	6 months; Walking exercises according to metronomes	low intensity
		62	75±6	41	21				
Alexandra2017	United States	10	58.2±17.2	7	3	CE	ID	4 months; 3/week ； Perform cycling exercises during dialysis	RPE12-14
		10	52.5±15.4	7	3				

Study	County	Croup (n)	Age (years)	Gender		Interventions			
				female	male	Type	Period	Prescription	Intensity level
Nataly2017	Brazil	29	48.86±12.97	17	12	IMT	ID	12 weeks； 3/week; Practice 12 times at first for 30 minutes each time; The last 12 exercises last 40 minutes each	15 cmH2O- 20 cmH2O
		12	52.67±14.61	7	5				
Clara2014	United Kingdom	27	52±14.5			CE	ID	24weeks； 3/week; Lasts10-30 minutes, with a goal of 60 minutes at the end	RPE12-14
		21	53±16.9			AT		24weeks； 3/week; Use the pedometer to walk10,000 steps per day	RPE12-14
Jeong2019	United States	29	53.7±11.4	17	12	CE	ID	12months; Perform cycling exercises during dialysis	RPE12-14
		34	54.4±12.3	18	17				
Hanan2015	Egypt	18	---	8	10	AT‘	ID	8weeks； 3/week; ROM exercises are performed on dialysis for 15 min	low intensity
		12	---	6	6				
Pellizzaro2013	Brazil	11	43±13.8	8	3	IMT	ID	three sets of 15 knee extension repetitions, resting for 60 s in between	50% of PImax
		14	48.9±10.1	7	7	RT		8weeks； Patients take three sets of 15 inhalations at the device nozzle and rest for 60 seconds	50% of this 1MR

Study	County	Croup (n)	Age (years)	Gender		Interventions			
				female	male	Type	Period	Prescription	Intensity level
		14	51.9±11.6	8	6				
Shota2014	Japan	6	68（61-78）	3	3	AT	ND	12weeks； 3/week; The patient stands up in 3 seconds and sits down in 3 seconds. 5 groups and 4 short breaks. Lasts 15 minutes	---
		11	69（64-79）	8	3			12weeks ； 3/week; Receive passive stretching of the shoulders, hips, knees, and ankles. Each joint and each direction took 30 seconds, and the total duration of 1 session was about 15 minutes	
Medeiros2018	UK	10	45.5(39.02-51.98)	---	---	IMT	ALL	8weeks； 2/week;； Three groups of 30 transphrasal respiratory patterns with a 1-minute break between groups	50% MIP load
		11	47.33（39.59-55.07）	---	---				
Kenneth2010	USA	7	60.8±3.2	3	4	CE	ID	4months; 3/week;； It starts at first with 5 minutes until it can last for 45 minutes	RPE12-14
		8	59±4.9	3	5				
Ouzouni2009	Greece	19	47.4±15.7	14	5	CT	ID	10months; 3/week; ； Aerobic and	RPE13-14

Study	County	Croup (n)	Age (years)	Gender		Interventions			
				female	male	Type	Period	Prescription	Intensity level
								strength exercises (30 minutes of cycling and 30 minutes of reinforcement and flexibility exercises, cycling time gradually increases until 60 minutes + strength training sessions include multiple sets of repetitions of the abdomen and lower extremities).	
		14	50.5±11.7	13	1				
DESAI2018	UK.	18	64.6±16.6	14	4	CE	ID	4months; 3/week; ; Each workout consists of three phases: warm-up, conditioning, and cooling.	RPE13-15
		21	70.1±13	15	6	CT			
Bechir Frih2017	United States	13	64.2±3.4	14	4	CT	ID	4months; 3/week; Lasts for 60 minutes (10 minutes warm up + aerobic and anaerobic combination + 10 minutes cool down).	(1-RM) starts at 50% and increases by 5% per month.
		21	65.2±3.1	15	6				
Rodrigo2019	Brazil	19	49.4±15.9	9	10	BFRT	ID	12weeks ; 3/week; ; 20 minutes of blood flow restriction exercise	RPE12-13
		20	59.8±16.1	11	9	CE		12weeks ; 3/week; ; 20 minutes of	RPE12-13

Study	County	Croup (n)	Age (years)	Gender		Interventions			
				female	male	Type	Period	Prescription	Intensity level
								pedaling exercises	
		20	48.2±13.6	9	11				
Taohaiyan2017	China	50	52±6	29	21	CE	ID	12weeks; 5-10 minutes warm up + 15 minutes to tidy up exercise + 20-30 minutes to pedal bike exercises	30%-60%VO_2Peak
		50	54±8	27	23				
Liaoli2018	China	33	49.76±5.53	18	15	AT	ND	12weeks; 3-5/week; ; On foot or by bicycle, within 60 minutes	
		32	50.14±5.68	20	12				
Wanghui2017	China	40	49.3±4.4	21	19	AT	ND	12weeks; 3-5/week; ; In the form of walking or cycling, etc., the duration is greater than 30 minutes	Slightly sweaty, slightly tired
		40	49.1±4.5	22	18				
Tangdongxing2015	China	33	41.4±10.5	17	16	CT	ND	10weeks; Walk or jog for 45 to 60 minutes a day on Mondays, Wednesdays and Fridays; Resistance exercise training is exercise for 20 to 30 minutes a day every Tuesday and Saturday	RPE13-15
		33	38±8.4	17	16				

Study	County	Croup (n)	Age (years)	Gender		Interventions			
				female	male	Type	Period	Prescription	Intensity level
Zhanghongmei2015	China	40	52.79±12.91	21	19	MBT	ND	3 months; Intensive study, 1 time/week; Initially 15 to 20 min each time, then add 5 min every 2 weeks, reach 40 ~ 45 min at 12 weeks, and practice by yourself for 3-5 times/week	60-70%HRmax
		40	50.34±14.47	22	18				
Liping2016	China	41	48.8±7.8	19	27	AT		6 months; Multiple forms of aerobic exercise; Lasts 20-30 minutes	60-70%HRmax
		43	53.4±14.6	17	26				
Huangliu2015	China	60	---	36	24	CE	ND	12weeks； 3/week; 5 to 10 minutes of warm-up exercises, 20 minutes of soft gymnastics, walking, stretching activities and 5 to 10 minutes of finishing exercises + 30 minutes of cycling exercises	I feel slightly tired, sweaty, no palpitations and wheezing
		60	---	31	29				
Cindy1995	United States	36	---	---	---	CE	ID	8weeks； 3/week; Lasts20-30 minutes	Wheel drag increases in phases, with each phase lasting 2 minutes. The working speed

Study	County	Croup (n)	Age (years)	Gender		Interventions			
				female	male	Type	Period	Prescription	Intensity level
									increases by 150 kpm/min per stage.
		35	---	---	---				
Marchesan2016	Brazil	8	63.2±3.5	6	2	CT	ID	The ankle exercise is performed with the help of a step, and the patient's toes are supported for flexion and extension exercises. The program takes place 3 times a week for 24 weeks. The duration of each workout starts at 45 minutes and gradually increases over time.	60-70%HRmax
		7	64.7±5.2	5	2				
Jamshidpour2019	Iran	13	64.93±7.79	---	---	CT	ID	8weeks； 3/week; Engage in 20 to 45 minutes of static cycling and lower limb resistance exercises	RPE13-15<br>60% 3RM
		13	58.46±11.85	---	---				
Mei Huang2019	China	16	43.81±10.25	12	4	CT	ID	24weeks； 15 min stretch + 5 min warm up + 30 min combined aerobic and impedance exercise + 5 min cool down	RPE12-14
		16	37.83±10.31	11	5				

Study	County	Croup (n)	Age (years)	Gender		Interventions			
				female	male	Type	Period	Prescription	Intensity level
Mei-Ling2020	Taipei	30	57.87±13.21	11	19	CT	ID	12 weeks, including warm-up, main and cooling exercise phases, lasting 30 minutes	RPE12-14
		32	53.91±12.6	17	15				
Fan Zhang2020	China	43	60（51-66）	27	26	RT	ID	2 times a week in weeks 1-4, 8-10 times in 2 sets of each action, 3 times a week for the next 8 weeks, and 3 groups of 11-12 weeks; 5 minutes of warm-up (no resistance to lift the arms and lower limbs), 30-40 minutes of exercise (including 20-30 movement minutes, rest between each group for 2-3 minutes) and 5 minutes of cooling (passive stretching of the lower limbs after exercise to promote recovery);	RPE10-13
		44	62（54-68）	26	18				
Anastasia2016	Greece	15	48±11.3	13	2	AT	ND	minutes 3 times a week for 4 months, with a warm-up period of 10 minutes, including stretching exercises. The patient then enters the pool for a swim, gradually increasing from 20 minutes at the beginning of the project to 40 minutes.	RPE13-14

Study	County	Croup (n)	Age (years)	Gender		Interventions			
				female	male	Type	Period	Prescription	Intensity level
								Relax for 10 minutes after each lesson	
		12	48.6±15.4	11	1				
Fuza2018	Brazil	8	61.5±9.91	---	---	WBVT	ND	12weeks ; 2-3/week ; The WBV training session lasts approximately 1 hour. , the exposure time of the vibratory session should not exceed a maximum of 30 minutes per day	---
		8	53.63±7.7		---	---			
Jo-Han Chang2016	Taiwan	21	54.2±15.2	13	8	MBT	ND	12weeks ; 10-15 minute warm-up exercises, followed by 40 minutes of 13 movement tai chi exercises, ending with a 5-10 minute cooldown.	Low strength
		25	54.6±12.7	17	8				
Nilsson2019	Norway	3	59.5 (55-67)	---	---	HICT	ID	16weeks; 2/week; Lasts for 5 minutes	Low to moderate intensity
		5	57 (25-68)	---	---	MICT		16weeks; 2/week; Lasts for 5 minutes	Low to moderate intensity
		3	67 (51-69)	---	---				
Luoxianglian2021	China	36	48.83±9.01	11	25	MBT	ND	12weeks ; 2/week ; The content includes 3 parts: warm-up exercises,	Low strength

Study	County	Croup (n)	Age (years)	Gender		Interventions			
				female	male	Type	Period	Prescription	Intensity level
								smiley yoga exercises, and meditation and relaxation	
		37	47.76±9.07	12	25				
Jiaxijiao2020	China	32	66.33±5.78	14	18	MBT	ND	6months; 5/week duration 45 minutes	Low strength
		32	66.42±6.21	12	20				
Tajmohammad2021	Iran	26	45.76±2.11	12	14	IMT	ND	3months; The breathing exercise program includes the use of IS equipment	ND
		26	48.12±3.01	10	16				
Argyro2022	Greece	21	66.04±15.35	5	16	CE	ID	7months; Duration 60 min; Pedal at 45 rpm for the first 10 minutes, then pedal at 60 rpm on a bedside bike dynamometer	60% maximum power
		23	68.26±11.07	13	10				
CaiShanShan2020	China	59	57.62±3.3	33	26	CT	ND	3months； Increased aerobic resistance exercises are given	ND
		58	57.53±3.21	30	28				
Yuxintao2021	China	29	52.56±12.47	13	16	CT	ID	6months; 3/week duration 60 minutes (40 minutes of aerobic exercise + 20 minutes	RPE13-14

Study	County	Croup (n)	Age (years)	Gender		Interventions			
				female	male	Type	Period	Prescription	Intensity level
								of resistance exercise)	
		30	53.91±14.69	16	14				
Yexiaoshuang2022	China	16	69.87±6.13	10	6	CT	ND	12weeks； 3/week； Bicycle + resistance training; Exercise time (T): 15-30 minutes min; Increase training intensity based on patient tolerance (see table Increasing Pedaling Resistance, Time, and Limb Load Intensity)	RPE11-13
		15	73.06±6.13	11	4				
Francini2021	China	20	55.9±15.42	14	6	CE	ID	12weeks； 2/week； Lasts for 30 minutes (5 minutes to warm up, 20 minutes to high strength, 5 minutes to cool down)	strong and very strong
		19	52.31±15.38	9	10				
Sharlene2014	United Kingdom	8	53.8±13.5	6	2	CT	ALL	12months; 3/week twice supervision; Once as a home exercise program; 40 minutes of training each time	80% heart rate reserve
		10	53.3±12.9	9	1				
Vicent2015	Spain	11		4	7	CT	ND	12weeks； 2/week； Lasts 45-50	40-50-60 rpm

Study	County	Croup (n)	Age (years)	Gender		Interventions			
				female	male	Type	Period	Prescription	Intensity level
								minutes	
		11		7	4			123	
SeEvangelia2018	Spain	8	61.8±13	4	4	CT	ID	20weeks；3/week；Duration up to 30 minutes	RPE13-15
		9	68.3±15.6	6	3	VRT		20weeks；3/week；Duration up to 30 minute；Exercise using a VR software program	
Kirsten2009	Australia	25	52.3±10.9	10	5	CE	ID	6months; 3/week；Complete at least 15 minutes per workout for the first 2 weeks, progress to 30 minutes per workout at week 12, and progress to 45 minutes by week 24	RPE12-13
		25	52.1±13.6	11	4	AT		6months; 3/week；Unsupervised walking	RPE12-13
		16	51.3±14.4	8	8			123	
Danielle2014	United Kingdom	9	48±18	7	2	RT	ID	12weeks；3/week；Increased load with increased athleticism	RPE15
		10	58±15	6	4			123	

Study	County	Croup (n)	Age (years)	Gender		Interventions			
				female	male	Type	Period	Prescription	Intensity level
Clara2018	Brazil	28	54.49±11.97	20	8	RT	ID	12weeks； 3/week; Lasts 40-50 minutes; A total of 11 exercises were used for progressive resistance training,	15-20RM
		24	57.1±16.2	15	9				
Manfredini2015	Italy	28	66±14	20	8	AT	ND	Home exercise program with 10 minutes of walking time twice a day to maintain metronome use at home	60% threshold
		26	68±13	15	11				
Renata2016	Brazil	20	54.65±19.93	---	---	MEST	ID	8weeks； 3/week 6 Pulse width within 350 microseconds at 50 Hz for 2 seconds (if tolerable), with a 10-second break	50 Hz
		20	46.4±15.43	---	---				
Rafael2012	Brazil	12	56.9±14.8	9	3	Ct	ID	10weeks20 minutes of aerobic training on the circulatory dynamometer + resistance training of the hamstrings and quadriceps groups and arm muscles without vascular access	RPE13-14
		12	55.8±18.3	12	0			10week ； Resistance exercises are performed using elastic bands, healing balls and dumbbells, and ankles	15RM

Study	County	Croup (n)	Age (years)	Gender		Interventions			
				female	male	Type	Period	Prescription	Intensity level
Luiza2021	Brazil	47	46.65±9.46	33	14	RT“	ID	8weeks； 3/week； Each session lasts 30 minutes. Resistance exercises with tibias are performed on lower extremity muscle groups (quadriceps, adductor, abductor, and hip flexors), initially using 50% of the force work, determined by the maximum isometric voluntary contraction of the quadriceps.	50%
		46	44.91±8.77	26	20			Passive stretching of hamstring, hip abductor and adductor, and triceps surae. Each muscle group was repeated for 3 times, each time for 20 seconds, with a rest of 30 seconds between each repetition.	
Marchesan2013	Brazil	11	---	---	---	AT	ND	17weeks； 3/week； Duration gradually increased (20 to 45 minutes)	---
		11	---	---	---				
Thomas20056	United States	12	44±9	7	5	CE	ID	6 months; 3/week ； Duration gradually increased (20 to 45 minutes)	---
		12	39±9	8	4				
Esteve	Spain	13	65.7±12.8	---	---	MEST	ID	12weeks;2-3/week Conditioning procedure for the first week (25 minutes, 8 Hz,	---

Study	County	Croup (n)	Age (years)	Gender		Interventions			
				female	male	Type	Period	Prescription	Intensity level
								contraction 1.5 seconds, phase 25 minutes, relaxation 1.5 seconds); one week of aerobic endurance (28 minutes, 60 Hz, contraction 1.5 seconds, phase 8 seconds, relaxation 0.75 seconds); 2 weeks of rehabilitation - muscular atrophy (30 minutes, 25-40 Hz, contraction for 2 seconds, phase 4 seconds, relaxation for 1 second); 2 weeks of convalescent hypertrophy (33 minutes, 55 Hz, contraction 1.5 seconds, phase 6 seconds, relaxation 1 second); 3 weeks of muscle enhancement (35 minutes, 9 peaks: 275 Hz, 7 seconds, relaxation 1.5 seconds), last 3 weeks of strength endurance (38 minutes, 90 Hz,	
		7	71.6±12.1	---	---				
TOUSSAINT	Australia	9	67（60-83）	5	4	CE	ID	3 months; Duration 30 minutes	---
		10	70（28-77）	5	6				
Misa Miura2017	Japan	8	70.0 ± 3.1	5	3	CE	ID	16weeks；3/week; Lasts 60 minutes;	RPE11-13
		7	73.9± 7.8	4	3	AT		16weeks；3/week; Lasts 60 minutes;	RPE11-13

Study	County	Croup (n)	Age (years)	Gender		Interventions			
				female	male	Type	Period	Prescription	Intensity level
OLIVEROS2011	Chile	5	46±13	5	0	CT		16weeks； 3/week; These include warm-up periods and aerobic exercise using standing cycles	40-60%HRmax
		6	52.2±17	3	3				
Rosangela2021	Brasil	10	53.3±18.5	7	3	CT	ID	4months; 2/week Use a bicycle ergometer or mini bike (MEGAFORTH brand) for aerobic training + stretching + lower limb muscle strengthening + breathing exercises	30% da RM
		23	54.96±15.97	16	7				

Appendix D. Risk of bias assessment

Study ID	D1	D2	D3	D4	D5	Overall
Marieke2005	+	+	+	+	+	+
Petr Dobsak2011	!	!	+	+	+	!
Stephanie2016	+	+	+	+	+	+
Tadashi2018	+	!	+	+	+	!
Molsted2004	+	+	!	+	!	!
Vince2002	+	+	!	+	+	!
Pomidori2016	!	+	!	+	+	!
Yongyao2014	!	!	+	+	+	!
Petraki2008	!	!	!	+	!	!
Flavia Baria2014	!	!	+	+	+	!
Kouidi2009	!	!	!	+	+	!
Deligiannis1999	!	+	!	+	!	!
Koufaki2002	!	!	!	+	!	!
Konstantinidou200	!	!	+	+	+	!
Mustata2011	+	!	+	+	+	!
Tsuyuki2003	!	!	+	+	!	!
Reboredo2018	!	!	!	+	+	!
Kirsten2010	!	!	!	+	+	!
Cheema2007	+	+	+	+	+	+
Bobby2007	!	!	!	+	+	!
Lucía2020	!	!	!	-	+	-
Antonio2019	+	!	+	+	+	!
Gordon2018	!	!	+	+	+	!
Fabio2016	+	!	!	+	+	!
Jociane2016	+	!	+	+	+	!
Rossella2018	!	!	!	+	+	!
Alexandra2017	!	!	!	+	+	!
Nataly2017	+	+	!	+	+	!
Clara2014	+	!	!	+	+	!
Jeong2019	!	!	!	+	+	!
Hanan2015	!	!	!	+	+	!
Pellizzaro2013	-	!	!	+	+	-
Shota2014	!	+	!	+	+	!
Medeiros2018	+	+	!	+	+	!
Kenneth2010	-	!	!	+	+	-
Ouzouni2009	!	!	!	+	+	!
DESAI2018	!	!	!	+	+	!
Bechir Frih2017	!	!	!	+	+	!
Rodrigo2019	+	!	+	+	+	!
Taohaiyan2017	+	+	+	+	+	!
Liaoli2018	+	!	+	+	+	!
Wanghui2017	-	!	!	+	+	-
Tangdongxing2015	!	!	+	+	+	!
Zhanghongmei2015	-	!	!	+	+	-
Liping2016	-	!	!	+	+	-
Huangliu2015	-	!	+	+	+	-
Cindy1995	!	!	!	+	!	!
Marchesan2016	!	!	+	+	+	!
Jamshidpour2019	+	!	+	+	+	!
Mei Huang2019	+	!	!	+	+	!
Mei-Ling2020	!	!	!	+	+	!
Fan Zhang2020	!	+	+	+	+	!
Anastasia2016	+	!	+	+	+	!
Fuza2018	+	+	+	+	+	!
Jo-Han Chang2016	-	!	+	+	+	-
Nilsson2019	+	+	!	+	+	!
Luoxianglian2021	+	!	+	+	+	!
Jiaxijiao2020	!	!	+	+	+	!
Tajmohammad2021	!	!	+	+	+	!
Argyro2022	+	!	+	+	+	!
CaiShanShan2020	!	!	+	+	+	!
Yuxintao2021	+	!	!	+	+	!
Yexiaoshuang2022	!	!	+	+	+	!
Francini2021	+	+	+	+	+	+
Sharlene2014	+	+	+	+	+	+
Vicent2015	!	!	!	+	+	!
SeEvangelia2018	+	!	+	+	+	!
Kirsten2009	+	!	!	+	+	!
Clara2018	!	!	!	+	+	!
Manfredini2015	!	!	+	+	+	!
Renata2016	!	!	+	+	+	!
Rafael2012	+	+	+	+	+	+
Luiza2021	+	!	!	+	+	!
Marchesan2013	!	!	!	+	+	!
Thomas20056	!	!	!	+	+	!
Wangtingting2022	!	!	+	+	+	!
Esteve2016	-	!	!	+	+	-
TOUSSAINT2008	!	+	+	+	+	!

\+ Low risk
! Some concerns
\- High risk

D1 Randomisation process
D2 Deviations from the intended interventions
D3 Missing outcome data
D4 Measurement of the outcome
D5 Selection of the reported result

(a)The data on the risk of bias assessment for each study.

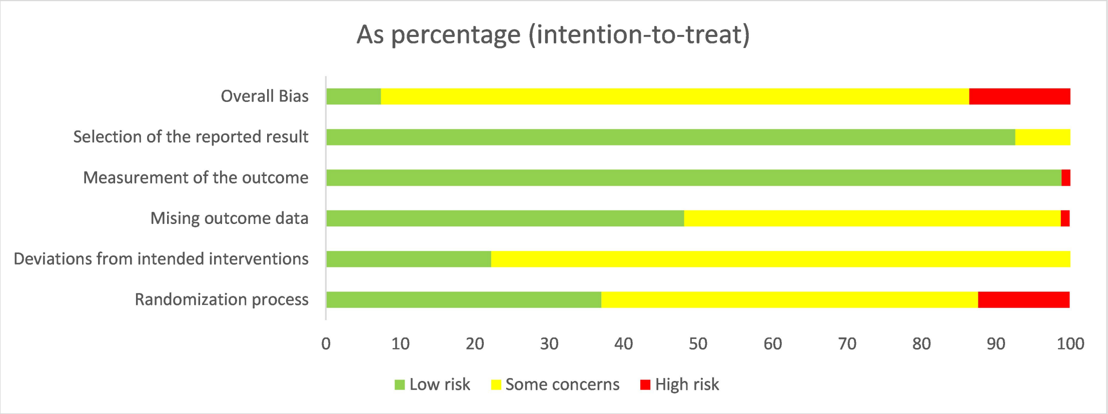

(b)Summary of bias risk assessment.

Appendix E. Comparisons with usual care.

a: Six-Minute Walkability. b: Peak oxygen uptake. c: systolic blood pressure. d: diastolic blood pressure .

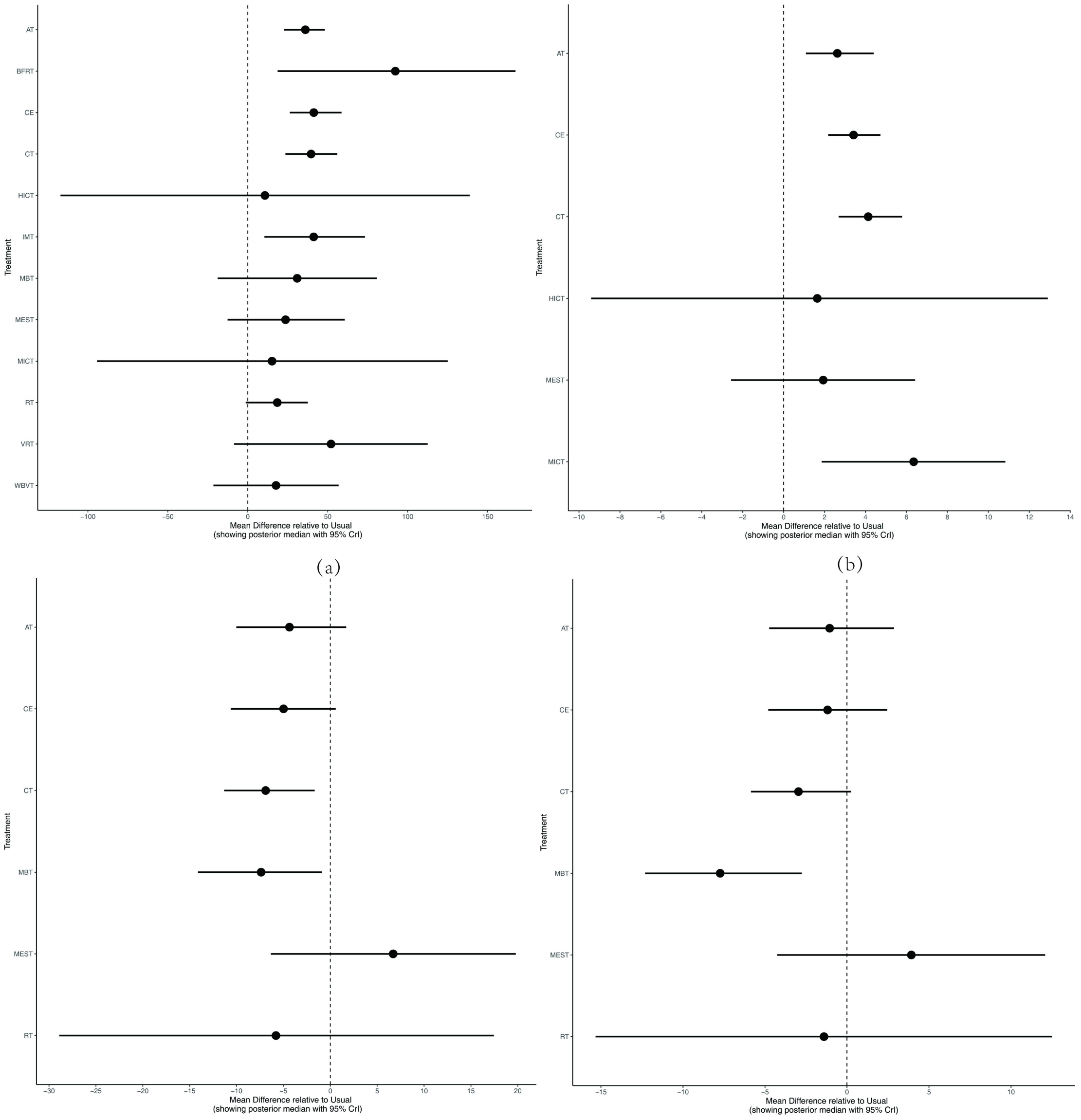

A: Forest plot of network meta-analysis of exercises compared with Usual care. RT= resistance training. CE= Cycle Dynamometers. MEST=Electrical muscle stimulation. IMT=Inspiratory muscle training. BFRT=Blood flow restriction exercise. HICT=High-intensity circuit training. MICT=Medium intensity continuous training. WBVT=Whole Body Vibration Training. VRT=Virtual Reality Training. MBT=Body and mind training.

Study	Experimental Total	Experimental Mean	Experimental SD	Control Total	Control Mean	Control SD	MD	95%−CI	Weight (common)	Weight (random)
**subgroup = AT VS Usual**										
Fabio Manfredini	28	50.00	94.78	26	−2.00	116.53	52.00	[−4.91; 108.91]	0.5%	1.6%
Luca Pomidori	22	39.00	105.09	20	3.00	101.01	36.00	[−26.35; 98.35]	0.4%	1.4%
Flavia Baria	10	103.70	86.94	9	15.00	77.43	88.70	[14.79; 162.61]	0.3%	1.1%
Antonio	20	38.25	88.44	19	−2.16	61.39	40.41	[−7.17; 87.99]	0.8%	2.0%
Fabio Manfredini−1	104	39.00	105.53	123	3.00	111.77	36.00	[7.69; 64.31]	2.2%	3.4%
Rossella Baggetta	53	33.00	80.67	62	−1.00	95.14	34.00	[1.87; 66.13]	1.7%	3.1%
Liaoli	33	59.05	31.87	32	1.90	35.11	57.15	[40.83; 73.47]	6.5%	4.4%
Wanghui	40	61.33	28.04	40	5.01	31.29	56.32	[43.30; 69.34]	10.2%	4.7%
Liping	41	22.21	40.79	43	1.10	42.83	21.11	[3.23; 38.99]	5.4%	4.3%
Samara Anastasia	15	66.80	127.80	12	−44.70	92.57	111.50	[28.28; 194.72]	0.3%	0.9%
Kirsten	25	49.00	135.71	16	21.00	152.63	28.00	[−63.78; 119.78]	0.2%	0.8%
Chengwenjiao	14	−8.39	66.37	40	2.26	90.79	−10.65	[−55.38; 34.07]	0.9%	2.2%
Marchesan	11	17.00	64.47	11	−6.60	63.46	23.60	[−29.86; 77.06]	0.6%	1.8%
Common effect model	416			453			44.65	[37.05; 52.25]	30.0%	−−
Random effects model							40.99	[27.97; 54.00]	−−	31.6%
Heterogeneity: $I^2 = 48.7\%$, $\tau^2 = 204.42$, $p = 0.025$										
Test for effect in subgroup (common effect): $z = 11.511$ ($p < 0.001$)										
Test for effect in subgroup (random effects): $z = 6.173$ ($p < 0.001$)										
**subgroup = CE VS Usual**										
Petr Dobsak	11	58.20	80.89	10	−12.60	77.08	70.80	[3.22; 138.38]	0.4%	1.3%
Yongyao Wu	32	33.00	120.58	22	22.00	119.85	11.00	[−54.22; 76.22]	0.4%	1.3%
Rodrigo Kohn	22	6.41	117.80	22	−11.10	104.37	17.51	[−48.26; 83.28]	0.4%	1.3%
Taohaiyan	50	49.00	23.58	50	23.00	26.51	26.00	[16.17; 35.83]	17.9%	4.9%
Huangliu	60	37.30	25.03	60	4.23	26.20	33.07	[23.90; 42.24]	20.6%	5.0%
Mei−Ling Yeh	30	55.70	96.43	32	−13.84	82.08	69.54	[24.82; 114.26]	0.9%	2.2%
Argyro A. Krase	21	63.80	93.96	23	1.10	76.56	62.70	[11.77; 113.63]	0.7%	1.9%
Kirsten	25	63.00	114.97	16	21.00	152.63	42.00	[−45.32; 129.32]	0.2%	0.8%
Wangtingting	25	139.80	59.94	25	15.52	63.61	124.28	[90.02; 158.54]	1.5%	2.9%
Common effect model	276			260			34.48	[28.13; 40.83]	43.0%	−−
Random effects model							51.81	[25.99; 77.63]	−−	21.6%
Heterogeneity: $I^2 = 77\%$, $\tau^2 = 987.73$, $p < 0.001$										
Test for effect in subgroup (common effect): $z = 10.640$ ($p < 0.001$)										
Test for effect in subgroup (random effects): $z = 3.933$ ($p < 0.001$)										
**subgroup = CT VS Usual**										
Vince	17	3.30	45.70	18	3.40	86.70	−0.10	[−45.66; 45.46]	0.8%	2.1%
Bechir Frih	11	60.50	33.61	10	−6.60	32.55	67.10	[38.79; 95.41]	2.2%	3.4%
Tangdongxing	33	58.90	89.33	33	15.00	75.05	43.90	[4.09; 83.71]	1.1%	2.5%
Marchesan	8	0.00	80.20	7	2.00	71.50	−2.00	[−78.77; 74.77]	0.3%	1.0%
Jamshidpour	15	89.44	89.09	13	7.21	90.94	82.23	[15.32; 149.14]	0.4%	1.3%
Mei Huang	16	42.62	50.80	16	−3.41	37.13	46.03	[15.20; 76.86]	1.8%	3.2%
Yuxintao	29	33.30	92.59	30	−5.91	75.20	39.21	[−3.91; 82.33]	0.9%	2.3%
Yexiaoxia	16	24.67	22.59	15	0.75	26.03	23.92	[6.71; 41.13]	5.9%	4.4%
Esteve Simo	11	40.30	133.40	11	−3.10	117.03	43.40	[−61.47; 148.27]	0.2%	0.6%
Jonathan Myers	13	22.60	76.51	15	9.70	59.31	12.90	[−38.39; 64.19]	0.7%	1.9%
Common effect model	169			168			35.22	[24.18; 46.27]	14.2%	−−
Random effects model							36.77	[21.21; 52.34]	−−	22.6%
Heterogeneity: $I^2 = 31.2\%$, $\tau^2 = 189.43$, $p = 0.159$										
Test for effect in subgroup (common effect): $z = 6.249$ ($p < 0.001$)										
Test for effect in subgroup (random effects): $z = 4.631$ ($p < 0.001$)										
**subgroup = IMT VS Usual**										
Nataly Gurgel Campos	29	78.50	105.59	12	−50.00	94.04	128.50	[62.87; 194.13]	0.4%	1.3%
Cíntia O. Pellizzaro	11	64.90	100.04	14	0.30	117.15	64.60	[−20.61; 149.81]	0.2%	0.9%
Medeiros	12	8.25	92.29	12	−11.90	80.24	20.15	[−49.04; 89.34]	0.4%	1.2%
Tajmohammad	26	10.65	56.20	26	0.77	66.18	9.88	[−23.49; 43.25]	1.6%	3.0%
Common effect model	78			64			35.08	[9.06; 61.11]	2.6%	−−
Random effects model							51.94	[−3.61; 107.49]	−−	6.3%
Heterogeneity: $I^2 = 71.7\%$, $\tau^2 = 2181.11$, $p = 0.014$										
Test for effect in subgroup (common effect): $z = 2.642$ ($p = 0.008$)										
Test for effect in subgroup (random effects): $z = 1.832$ ($p = 0.067$)										
**subgroup = MEST VS Usual**										
Petr Dobsak	11	27.60	63.31	10	−12.60	77.08	40.20	[−20.48; 100.88]	0.5%	1.5%
Jociane Schardong	11	21.48	117.22	10	8.70	81.12	12.78	[−72.81; 98.37]	0.2%	0.9%
Renata Spósito	20	22.80	106.08	20	−2.80	62.68	25.60	[−28.40; 79.60]	0.6%	1.7%
Esteve	13	32.00	170.49	7	5.60	87.63	26.40	[−86.75; 139.55]	0.1%	0.5%
Common effect model	55			47			28.35	[−6.38; 63.08]	1.4%	−−
Random effects model							28.35	[−6.38; 63.08]	−−	4.6%
Heterogeneity: $I^2 = 0\%$, $\tau^2 = 0$, $p = 0.963$										
Test for effect in subgroup (common effect): $z = 1.600$ ($p = 0.110$)										
Test for effect in subgroup (random effects): $z = 1.600$ ($p = 0.110$)										
**subgroup = RT VS Usual**										
Bobby Cheema	24	16.70	40.50	25	−2.90	25.80	19.60	[0.50; 38.70]	4.7%	4.2%
Bobby Cheem	20	36.30	50.60	19	8.60	44.40	27.70	[−2.14; 57.54]	1.9%	3.2%
Cíntia O. Pellizzaro	14	30.40	74.71	14	0.30	117.15	30.10	[−42.68; 102.88]	0.3%	1.1%
Fan Zhang	43	2.95	86.97	44	−0.36	90.48	3.31	[−33.98; 40.60]	1.2%	2.7%
Danielle	9	39.00	98.14	10	64.00	50.86	−25.00	[−96.45; 46.45]	0.3%	1.2%
Clara Suemi	28	20.32	128.30	24	16.77	166.15	3.55	[−78.16; 85.26]	0.3%	0.9%
Common effect model	138			136			17.30	[3.32; 31.28]	8.9%	−−
Random effects model							17.30	[3.32; 31.28]	−−	13.3%
Heterogeneity: $I^2 = 0\%$, $\tau^2 = 0$, $p = 0.756$										
Test for effect in subgroup (common effect): $z = 2.426$ ($p = 0.015$)										
Test for effect in subgroup (random effects): $z = 2.426$ ($p = 0.015$)										
Common effect model	1132			1128			36.04	[31.87; 40.20]	100.0%	−−
Random effects model							37.93	[29.27; 46.60]	−−	100.0%

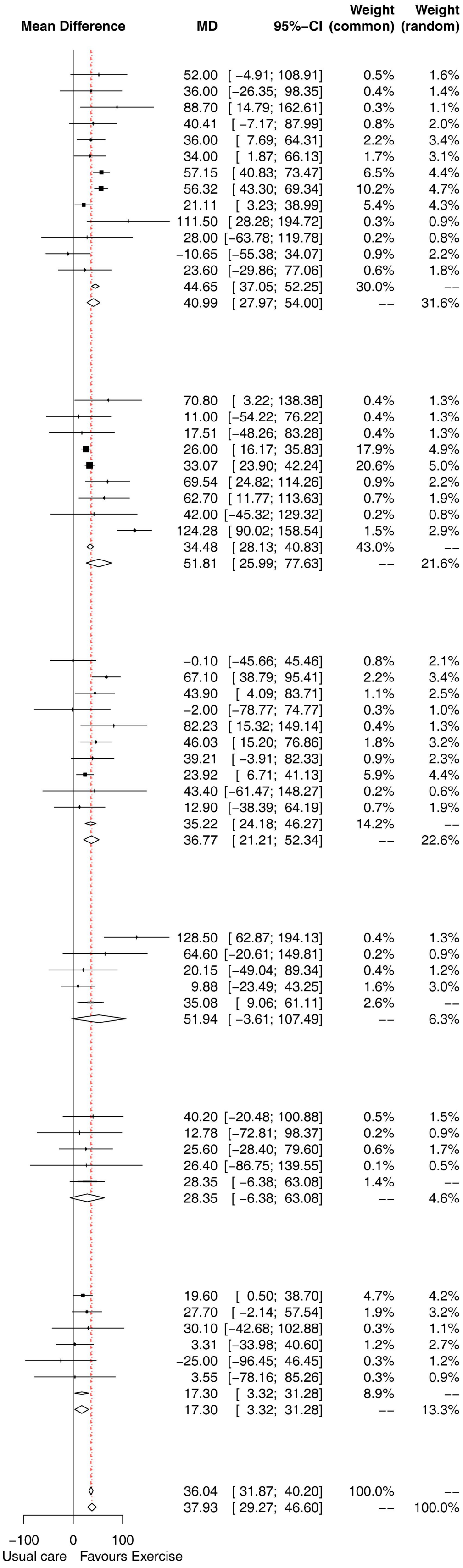

Heterogeneity: $I^2 = 53.6\%$, $\tau^2 = 371.48$, $p < 0.001$
Test for overall effect (common effect): $z = 16.967$ ($p < 0.001$)
Test for overall effect (random effects): $z = 8.577$ ($p < 0.001$)
Test for subgroup differences (common effect): $\chi^2_5 = 12.277$, df = 5 ($p = 0.031$)
Test for subgroup differences (random effects): $\chi^2_5 = 9.038$, df = 5 ($p = 0.108$)

a. Six-Minute Walkability

Study	Experimental Total	Experimental Mean	Experimental SD	Control Total	Control Mean	Control SD	MD	95%-CI	Weight (common)	Weight (random)
**subgroup = AT VS Usual**										
Stefan Mustata	10	2.40	3.94	10	0.70	16.84	1.70	[−9.02; 12.42]	0.2%	0.6%
Flavia Baria	8	2.30	4.52	9	0.70	6.44	1.60	[−3.65; 6.85]	0.8%	2.2%
Kazuo Tsuyuki	17	5.50	4.85	12	−0.50	6.01	6.00	[1.89; 10.11]	1.3%	3.2%
wanghui	40	2.15	2.17	40	0.00	2.51	2.15	[1.12; 3.18]	21.3%	10.8%
Liaoli	33	2.15	2.17	32	0.00	2.51	2.15	[1.01; 3.29]	17.3%	10.4%
Common effect model	108			103			2.26	[1.52; 3.00]	41.0%	--
Random effects model							2.26	[1.52; 3.00]	--	27.2%
Heterogeneity: $I^2 = 0\%$, $\tau^2 = 0$, $p = 0.503$										
Test for effect in subgroup (common effect): $z = 5.977$ ($p < 0.001$)										
Test for effect in subgroup (random effects): $z = 5.977$ ($p < 0.001$)										
**subgroup = CE VS Usual**										
Evangelia Kouidi	24	5.54	5.08	20	−0.33	3.95	5.87	[3.20; 8.54]	3.2%	5.7%
Pelagia Koufaki	18	2.99	6.20	15	−0.70	4.80	3.69	[−0.07; 7.45]	1.6%	3.7%
Erasmia	12	2.80	5.16	15	−0.50	4.75	3.30	[−0.48; 7.08]	1.6%	3.6%
Maycon 1	12	3.20	6.38	12	−2.90	6.76	6.10	[0.84; 11.36]	0.8%	2.2%
Gordon McGregor	17	2.46	6.66	18	−0.37	5.27	2.83	[−1.16; 6.82]	1.4%	3.3%
Evangelia J	30	5.00	6.22	29	−0.20	4.40	5.20	[2.46; 7.94]	3.0%	5.5%
Cindy	36	2.50	4.41	35	0.90	3.25	1.60	[−0.20; 3.40]	7.0%	8.2%
Francini	20	3.13	6.23	19	−0.18	4.52	3.31	[−0.09; 6.71]	1.9%	4.2%
Thomas	12	3.10	5.41	12	−1.10	6.64	4.20	[−0.65; 9.05]	1.0%	2.5%
Common effect model	181			175			3.54	[2.51; 4.56]	21.5%	--
Random effects model							3.78	[2.46; 5.10]	--	38.8%
Heterogeneity: $I^2 = 19.6\%$, $\tau^2 = 1.21$, $p = 0.269$										
Test for effect in subgroup (common effect): $z = 6.762$ ($p < 0.001$)										
Test for effect in subgroup (random effects): $z = 5.621$ ($p < 0.001$)										
**subgroup = CT VS Usual**										
Marieke	54	4.60	5.67	53	−1.10	4.55	5.70	[3.75; 7.65]	6.0%	7.7%
M Petraki	22	4.60	5.67	21	−1.10	4.55	5.70	[2.63; 8.77]	2.4%	4.8%
Erasmia	10	3.90	5.47	15	−0.50	4.75	4.40	[0.24; 8.56]	1.3%	3.2%
Stavroula Ouzouni	19	4.40	5.35	14	−0.20	3.50	4.60	[1.58; 7.62]	2.5%	4.9%
CaiShanShan	59	2.48	2.71	58	0.28	2.59	2.20	[1.24; 3.16]	24.5%	11.0%
Sharlene	8	2.90	5.57	10	−2.10	4.81	5.00	[0.12; 9.88]	0.9%	2.5%
Common effect model	172			171			3.28	[2.51; 4.06]	37.5%	--
Random effects model							4.31	[2.71; 5.91]	--	34.0%
Heterogeneity: $I^2 = 65.9\%$, $\tau^2 = 2.00$, $p = 0.012$										
Test for effect in subgroup (common effect): $z = 8.303$ ($p < 0.001$)										
Test for effect in subgroup (random effects): $z = 5.294$ ($p < 0.001$)										
Common effect model	461			449			2.92	[2.44; 3.39]	100.0%	--
Random effects model							3.61	[2.76; 4.46]	--	100.0%

Heterogeneity: $I^2 = 42.8\%$, $\tau^2 = 1.46$, $p = 0.023$
Test for overall effect (common effect): $z = 12.047$ ($p < 0.001$)
Test for overall effect (random effects): $z = 8.325$ ($p < 0.001$)
Test for subgroup differences (common effect): $\chi^2_2 = 5.261$, df = 2 ($p = 0.072$)
Test for subgroup differences (random effects): $\chi^2_2 = 7.535$, df = 2 ($p = 0.023$)

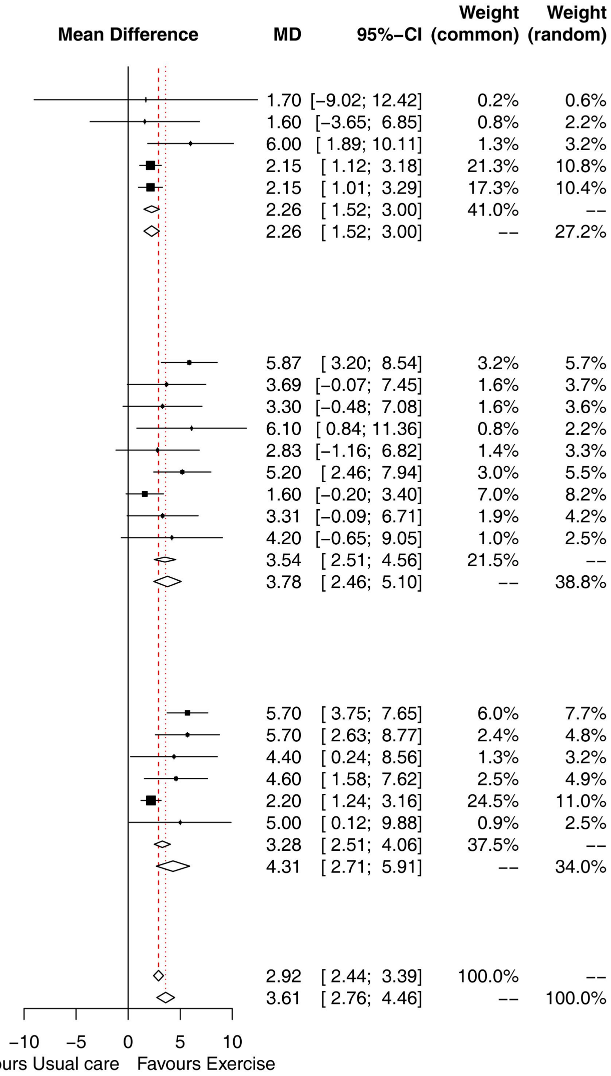

b. VO_2Peak

Study	Experimental Total	Experimental Mean	Experimental SD	Control Total	Control Mean	Control SD	MD	95%-CI	Weight (common)	Weight (random)
**subgroup = AT VS Usual**										
Asterios Deligiannis	10	−2.00	17.52	12	1.00	8.89	−3.00	[−14.97; 8.97]	1.0%	2.8%
Kazuo Tsuyuki	17	−1.70	18.34	12	−7.70	22.03	6.00	[−9.21; 21.21]	0.6%	1.9%
Kirsten	21	−2.00	30.61	22	−9.00	25.36	7.00	[−9.84; 23.84]	0.5%	1.6%
Hanan	18	−15.00	14.09	12	6.66	19.94	−21.66	[−34.68; −8.64]	0.9%	2.4%
Shota	6	−2.00	41.94	11	5.00	60.20	−7.00	[−55.91; 41.91]	0.1%	0.2%
Liping	41	−5.90	10.35	43	−1.00	8.65	−4.90	[−8.99; −0.81]	8.7%	7.4%
Chengyanjiao	14	−0.86	14.66	40	4.55	18.02	−5.41	[−14.90; 4.09]	1.6%	3.7%
Molsted	11	2.00	51.66	9	−0.50	37.12	2.50	[−36.49; 41.49]	0.1%	0.4%
Common effect model	138			161			−4.88	[−8.16; −1.60]	13.5%	--
Random effects model							−4.51	[−10.51; 1.48]	--	20.5%
Heterogeneity: $I^2 = 33.4\%$, $\tau^2 = 26.86$, $p = 0.162$										
Test for effect in subgroup (common effect): $z = -2.912$ ($p = 0.004$)										
Test for effect in subgroup (random effects): $z = -1.476$ ($p = 0.140$)										
**subgroup = CE VS Usual**										
Stephanie Thompson	8	−6.00	20.52	8	−1.00	21.70	−5.00	[−25.70; 15.70]	0.3%	1.1%
Kirsten	27	−9.00	22.00	22	−9.00	25.36	0.00	[−13.46; 13.46]	0.8%	2.3%
Gordon McGregor	17	5.68	25.61	18	3.84	25.71	1.84	[−15.17; 18.85]	0.5%	1.6%
Alexandra B. Cooke	10	−10.00	18.89	10	−0.30	8.52	−9.70	[−22.54; 3.14]	0.9%	2.5%
Jin Hee Jeong	29	0.10	25.56	34	−2.50	27.06	2.60	[−10.41; 15.61]	0.9%	2.4%
Kenneth	8	−2.90	13.89	9	24.40	14.97	−27.30	[−41.02; −13.58]	0.8%	2.3%
DESAI	18	5.00	20.23	21	7.00	21.61	−2.00	[−15.14; 11.14]	0.8%	2.4%
TOUSSAINT	9	−3.10	14.05	10	−2.60	22.04	−0.50	[−16.96; 15.96]	0.5%	1.7%
Antonio	20	1.50	14.81	19	2.63	15.94	−1.13	[−10.80; 8.54]	1.6%	3.7%
Common effect model	146			151			−4.50	[−9.02; 0.03]	7.1%	--
Random effects model							−4.63	[−10.90; 1.63]	--	20.0%
Heterogeneity: $I^2 = 43.6\%$, $\tau^2 = 40.44$, $p = 0.077$										
Test for effect in subgroup (common effect): $z = -1.946$ ($p = 0.052$)										
Test for effect in subgroup (random effects): $z = -1.449$ ($p = 0.147$)										
**subgroup = CT VS Usual**										
Marieke	54	−5.00	24.95	53	−4.00	24.06	−1.00	[−10.29; 8.29]	1.7%	3.8%
Stephanie Thompson	8	−5.00	27.60	8	−1.00	21.70	−4.00	[−28.33; 20.33]	0.2%	0.9%
Stig Molsted	11	2.00	51.67	8	0.50	43.78	1.50	[−41.54; 44.54]	0.1%	0.3%
VinCE	17	2.40	17.66	18	1.10	20.77	1.30	[−11.45; 14.05]	0.9%	2.5%
M Petraki	22	−8.30	15.25	21	−2.00	15.53	−6.30	[−15.50; 2.90]	1.7%	3.9%
Asterios Deligiannis	16	−9.00	15.72	12	1.00	8.89	−10.00	[−19.20; −0.80]	1.7%	3.9%
Ouzouni	19	−7.60	13.36	14	1.10	8.07	−8.70	[−16.05; −1.35]	2.7%	5.0%
Bechir Frih	21	−13.40	4.75	20	1.20	5.60	−14.60	[−17.79; −11.41]	14.3%	8.1%
Mei Huang	16	−8.50	14.76	16	−1.63	17.22	−6.87	[−17.98; 4.24]	1.2%	3.1%
Sharlene	89	−2.60	16.18	10	−7.20	19.12	4.60	[−7.72; 16.92]	1.0%	2.7%
Common effect model	273			180			−10.42	[−12.81; −8.03]	25.5%	--
Random effects model							−6.51	[−11.05; −1.96]	--	34.1%
Heterogeneity: $I^2 = 58.1\%$, $\tau^2 = 23.80$, $p = 0.011$										
Test for effect in subgroup (common effect): $z = -8.551$ ($p < 0.001$)										
Test for effect in subgroup (random effects): $z = -2.805$ ($p = 0.005$)										
**subgroup = MBT VS Usual**										
Zhanghongmei	40	−8.98	13.46	40	2.03	14.64	−11.01	[−17.17; −4.85]	3.8%	5.8%
Wurina	20	−8.19	14.63	29	−1.41	16.32	−6.78	[−15.52; 1.96]	1.9%	4.1%
Luolianxiang	37	−7.25	10.28	36	−1.38	12.39	−5.87	[−11.10; −0.64]	5.3%	6.5%
Jiaxijiao	32	−32.15	3.26	32	−25.95	4.19	−6.20	[−8.04; −4.36]	42.9%	9.0%
Common effect model	129			137			−6.53	[−8.17; −4.89]	54.0%	--
Random effects model							−6.53	[−8.17; −4.89]	--	25.4%
Heterogeneity: $I^2 = 0\%$, $\tau^2 = 0$, $p = 0.528$										
Test for effect in subgroup (common effect): $z = -7.800$ ($p < 0.001$)										
Test for effect in subgroup (random effects): $z = -7.800$ ($p < 0.001$)										
Common effect model	686			629			−7.15	[−8.36; −5.95]	100.0%	--
Random effects model							−6.10	[−8.47; −3.74]	--	100.0%

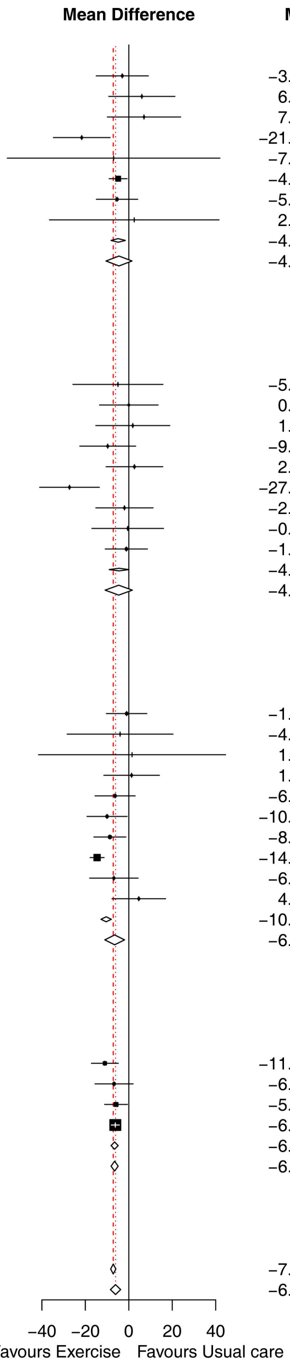

Heterogeneity: $I^2 = 49.4\%$, $\tau^2 = 15.25$, $p = 0.001$

Test for overall effect (common effect): $z = -11.633$ ($p < 0.001$)

Test for overall effect (random effects): $z = -5.066$ ($p < 0.001$)

Test for subgroup differences (common effect): $\chi^2_3 = 10.908$, df = 3 ($p = 0.012$)

Test for subgroup differences (random effects): $\chi^2_3 = 0.695$, df = 3 ($p = 0.874$)

c. SBP

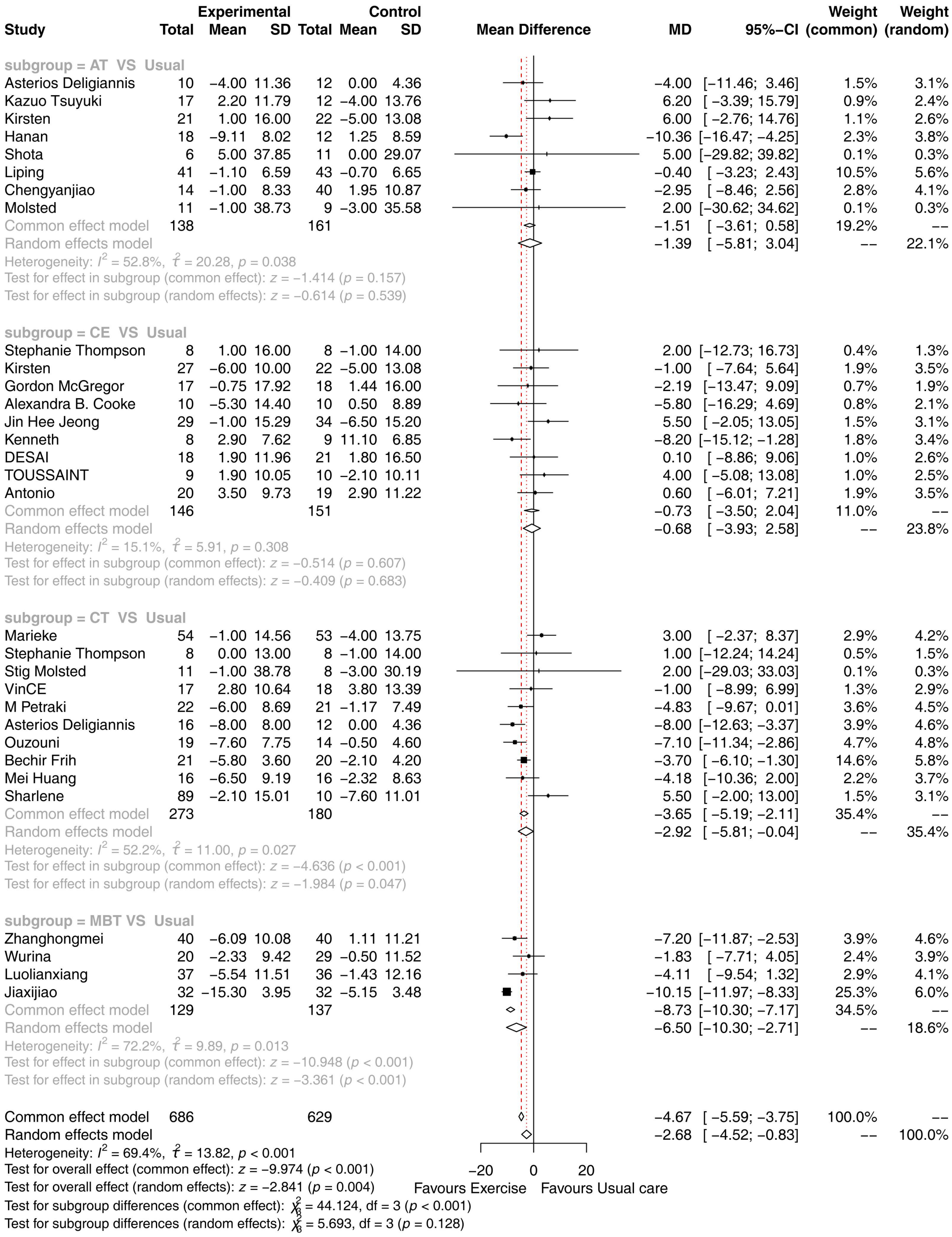

d. DBP

B:Forest plot of pair-wise meta-analysis of exercises compared with Usual care.

Data are reported as MDs with 95% CIs. AT=aerobic training. Usual=Usual care. CT=combined training. RT= resistance training. CE= Cycle Dynamometers. MEST=Electrical muscle stimulation. IMT=Inspiratory muscle training. BFRT=Blood flow restriction exercise. HICT=High-intensity circuit training. MICT=Medium intensity continuous training. WBVT=Whole Body Vibration Training. VRT=Virtual Reality Training. MBT=Body and mind training.

Appendix F. Model selection

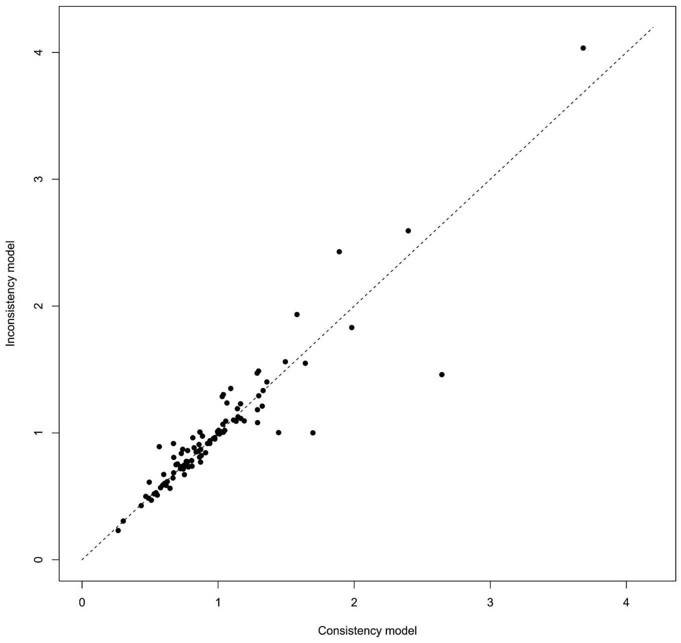

A(a)

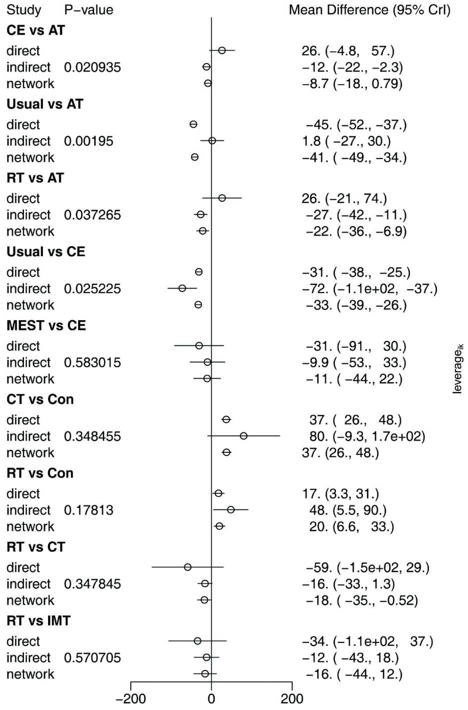

A(b)

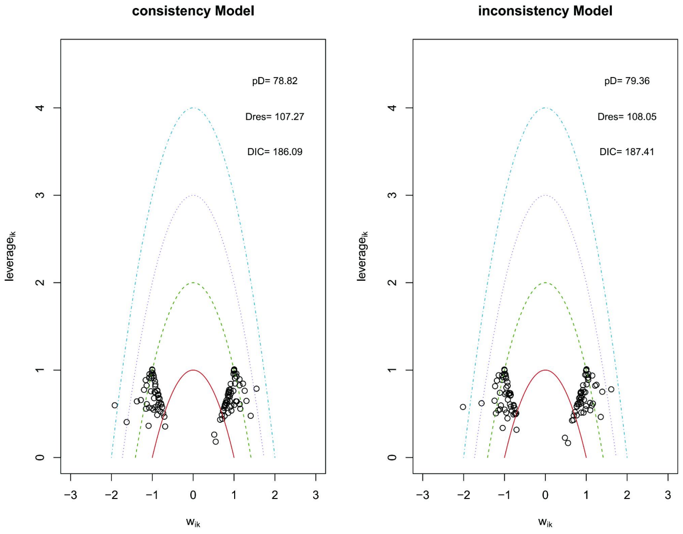

A(c)

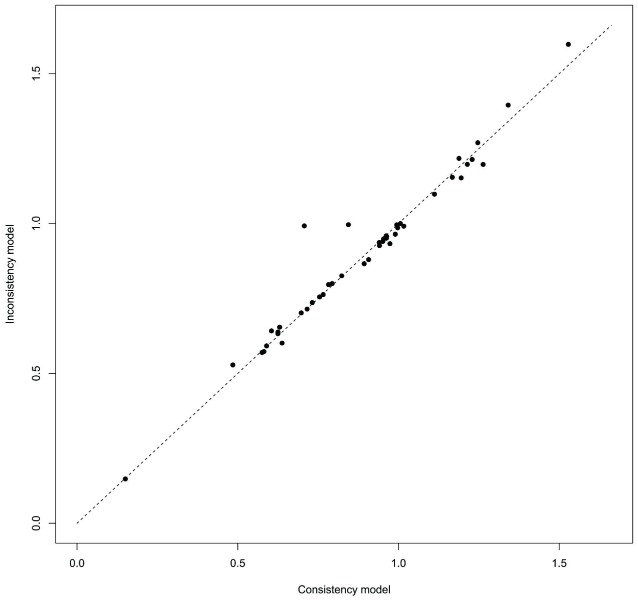

Inconsistency model
Consistency model
0.0
0.5
1.0
1.5

A(a)

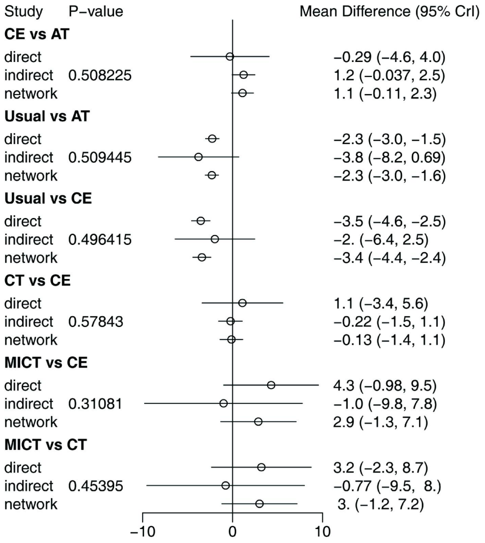

Study P−value
Mean Difference (95% CrI)
CE vs AT
direct −0.29 (−4.6, 4.0)
indirect 0.508225 1.2 (−0.037, 2.5)
network 1.1 (−0.11, 2.3)
Usual vs AT
direct −2.3 (−3.0, −1.5)
indirect 0.509445 −3.8 (−8.2, 0.69)
network −2.3 (−3.0, −1.6)
Usual vs CE
direct −3.5 (−4.6, −2.5)
indirect 0.496415 −2. (−6.4, 2.5)
network −3.4 (−4.4, −2.4)
CT vs CE
direct 1.1 (−3.4, 5.6)
indirect 0.57843 −0.22 (−1.5, 1.1)
network −0.13 (−1.4, 1.1)
MICT vs CE
direct 4.3 (−0.98, 9.5)
indirect 0.31081 −1.0 (−9.8, 7.8)
network 2.9 (−1.3, 7.1)
MICT vs CT
direct 3.2 (−2.3, 8.7)
indirect 0.45395 −0.77 (−9.5, 8.)
network 3. (−1.2, 7.2)
−10
0
10

A(b)

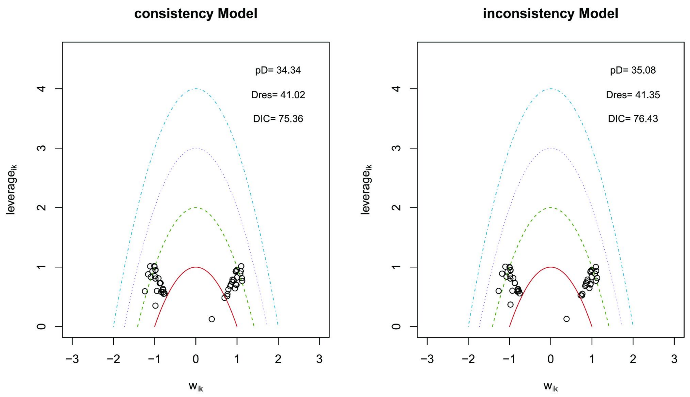

consistency Model
pD= 34.34
Dres= 41.02
DIC= 75.36
leverage_ik
w_ik
inconsistency Model
pD= 35.08
Dres= 41.35
DIC= 76.43
leverage_ik
w_ik

A(c)

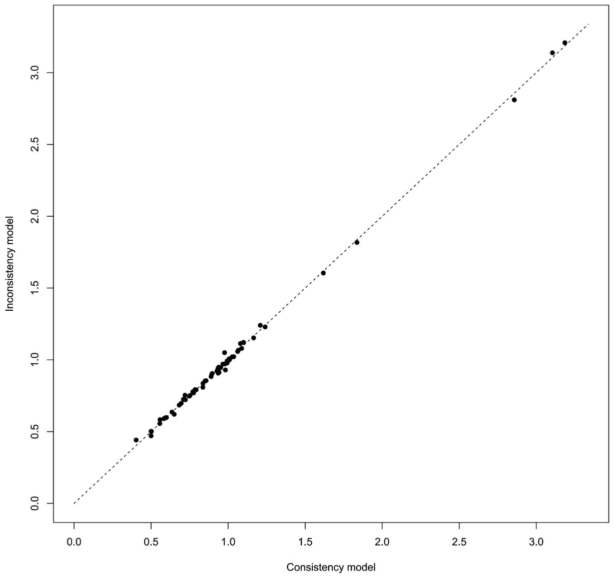
Inconsistency model
Consistency model
0.0
0.5
1.0
1.5
2.0
2.5
3.0

A(a)

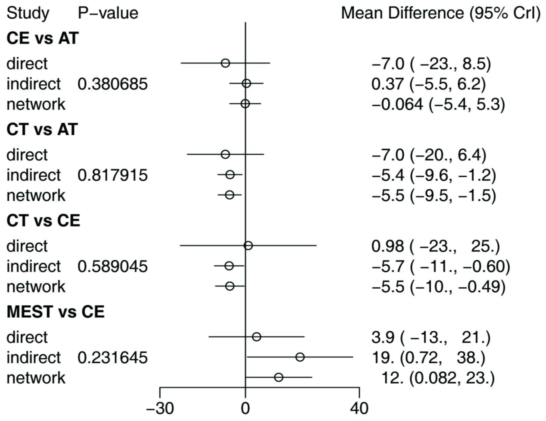
Study P−value
Mean Difference (95% CrI)
CE vs AT
direct −7.0 (−23., 8.5)
indirect 0.380685 0.37 (−5.5, 6.2)
network −0.064 (−5.4, 5.3)
CT vs AT
direct −7.0 (−20., 6.4)
indirect 0.817915 −5.4 (−9.6, −1.2)
network −5.5 (−9.5, −1.5)
CT vs CE
direct 0.98 (−23., 25.)
indirect 0.589045 −5.7 (−11., −0.60)
network −5.5 (−10., −0.49)
MEST vs CE
direct 3.9 (−13., 21.)
indirect 0.231645 19. (0.72, 38.)
network 12. (0.082, 23.)
−30
0
40

A(b)

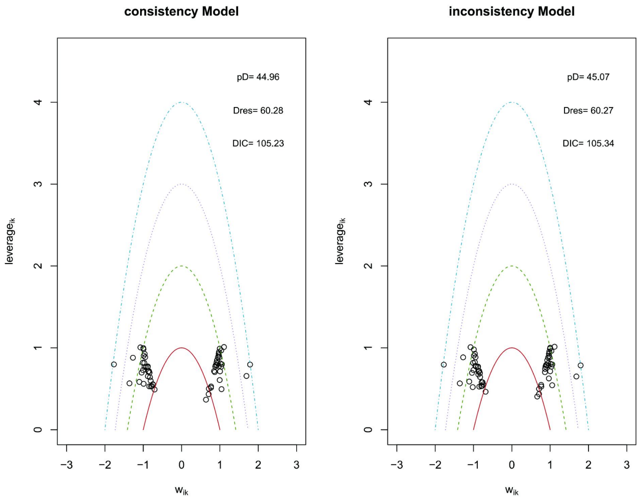
consistency Model
pD= 44.96
Dres= 60.28
DIC= 105.23
inconsistency Model
pD= 45.07
Dres= 60.27
DIC= 105.34
leverage
w

A(c)

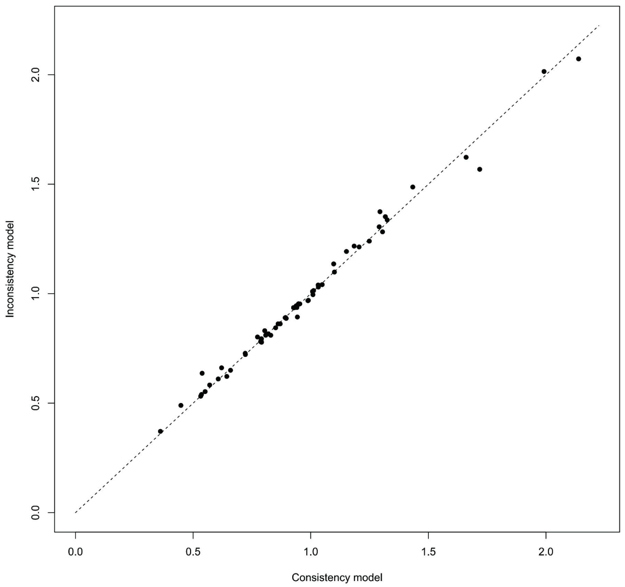

A(a)

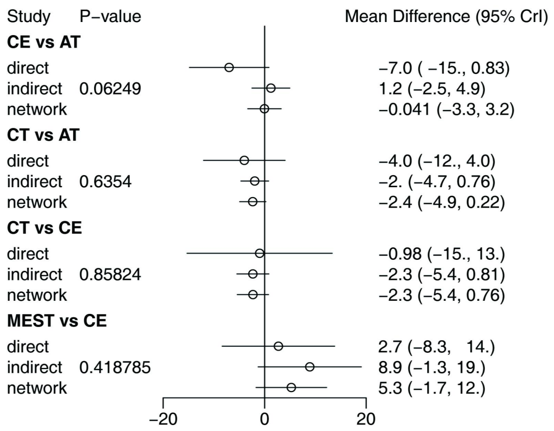

A(b)

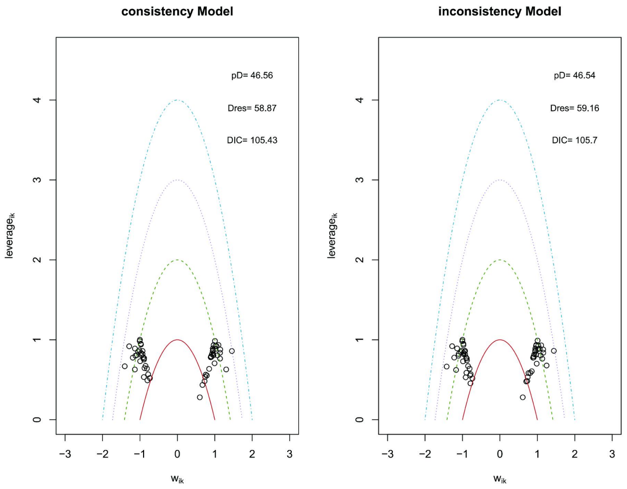

A(c)

A: Six-Minute Walkability. B: Peak oxygen uptake. C: systolic blood pressure. D: diastolic blood pressure.
a: posterior mean deviance contributions plot. The more the points are concentrated on the slants, the better the model fits.
b:Node splitting method. $P < 0.05$ indicates that the node is inconsistent.
c: Leverage plots. The smaller the DIC in the figure, the better the model fits.
AT=aerobic training. Usual=Usual care. CT=combined training. RT= resistance training. CE= Cycle Dynamometers. MEST=Electrical muscle stimulation. IMT=Inspiratory muscle training. BFRT=Blood flow restriction exercise. HICT=High-intensity circuit training. MICT=Medium intensity continuous training. VRT=Virtual Reality Training. MBT=Body and mind training.

Appendix G. Comprehensive assessment

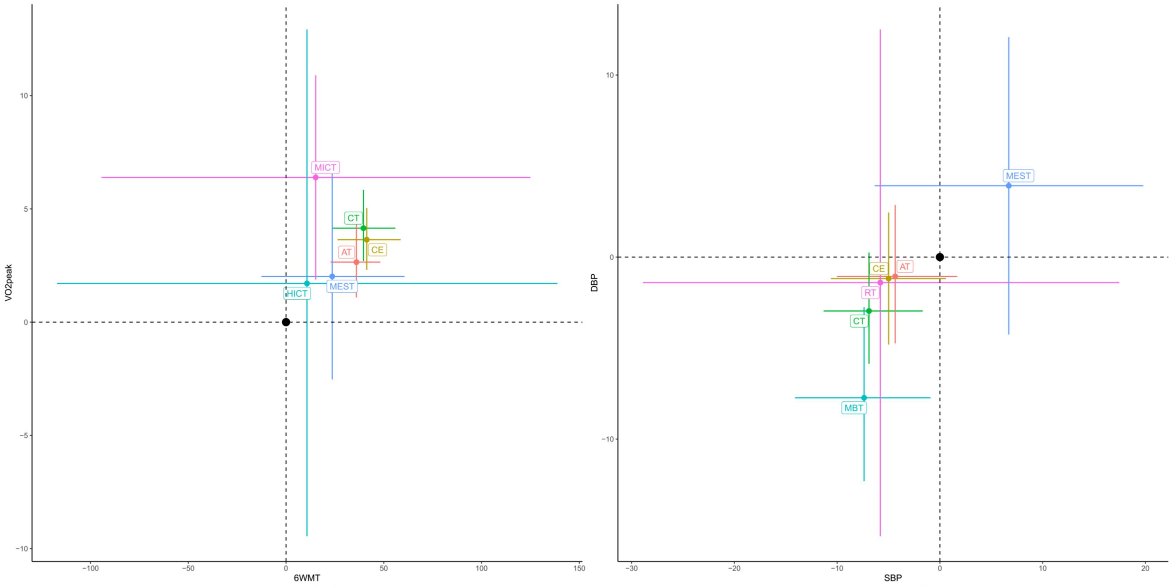

(a) (b)

Two-dimensional plots about efficacy in 6MWT with VO_2peak (a) and SBP with DBP (b)

Data are reported as MDs in comparison with Usual care, which is the black dot in the figure. Error bars are 95% CIs. Individual exercises are represented by different coloured nodes. MD=mean difference. CI=confidence interval. AT=aerobic training. CT=combined training. RT= resistance training. CE= Cycle Dynamometers. MEST=Electrical muscle stimulation. HICT=High-intensity circuit training. MICT=Medium intensity continuous training. MBT=Body and mind

training.

Appendix H. Sub-group analysis

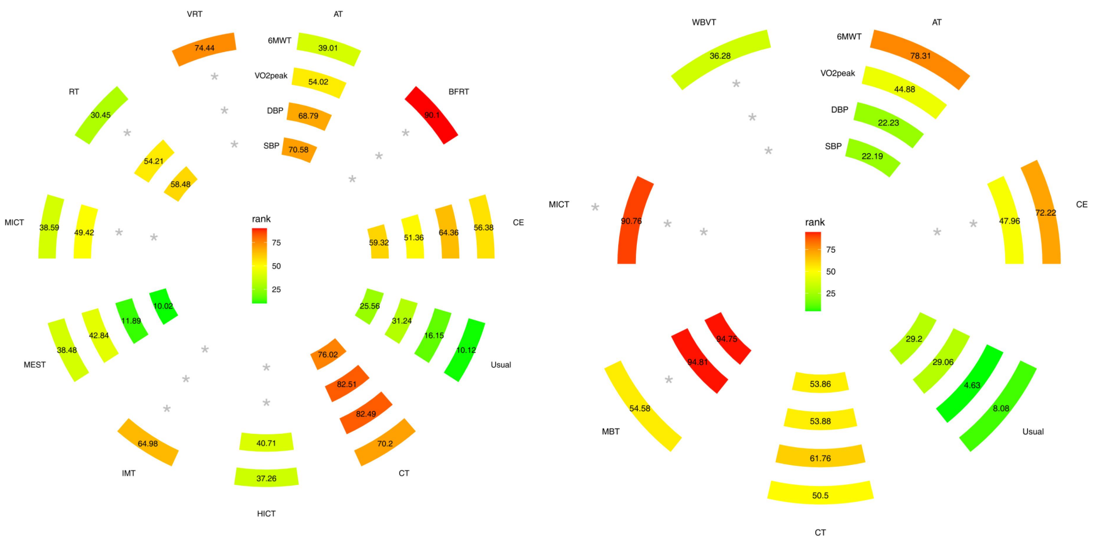

(a) (b)

Heat plots ranked according to the degree of relevant change in 6WMT, VO_2Peak, SBP, and DBP. The numbers reflect the SUCRA, ranked continuously from 0 to 100. A higher SUCRA indicates a more significant increase in 6WMT, VO2Peak. In the case of blood pressure parameters, a higher SUCRA suggests a more pronounced effect on lowering blood pressure.
a: exercise in dialysis environment. b: exercise in dialysis non-environment.
6MWT: Six-Minute Walkability. VO2Peak: Peak oxygen uptake. SBP: systolic blood pressure. DBP: diastolic blood pressure. AT=aerobic training. Usual=Usual care. CT=combined training. RT= resistance training. CE=Cycle Dynamometers. MEST=Electrical muscle stimulation. IMT=Inspiratory

muscle training. BFRT=Blood flow restriction exercise. HICT=High-intensity circuit training. MICT=Medium intensity continuous training. VRT=Virtual Reality Training. MBT=Body and mind training.

Appendix I. Sensitivity analysis.

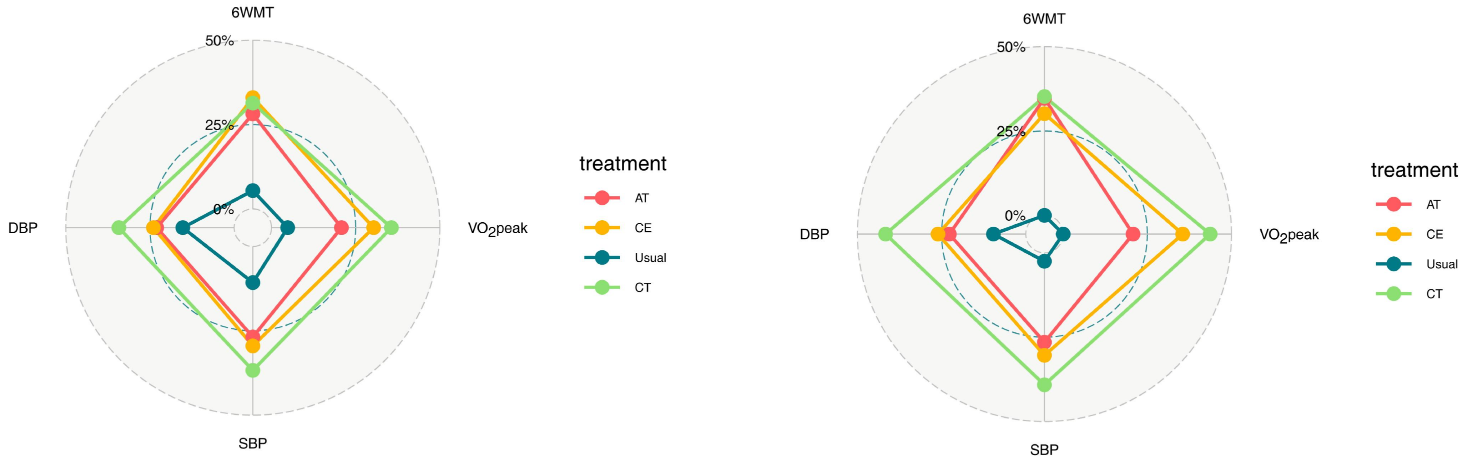

(a) (b)

The radar chart shows the three main exercise methods and Usual care with respect to the four outcomes of the comprehensive SUCRA. The greater the area enclosed by the curve, the greater the benefit of the intervention across all outcomes.
a: results from a network meta-analysis of all interventions. b: results from a network meta-analysis of only the three main exercises.
AT=aerobic training. Usual=Usual care. CT=combined training. CE= Cycle Dynamometers.

Appendix J. Regression analysis.

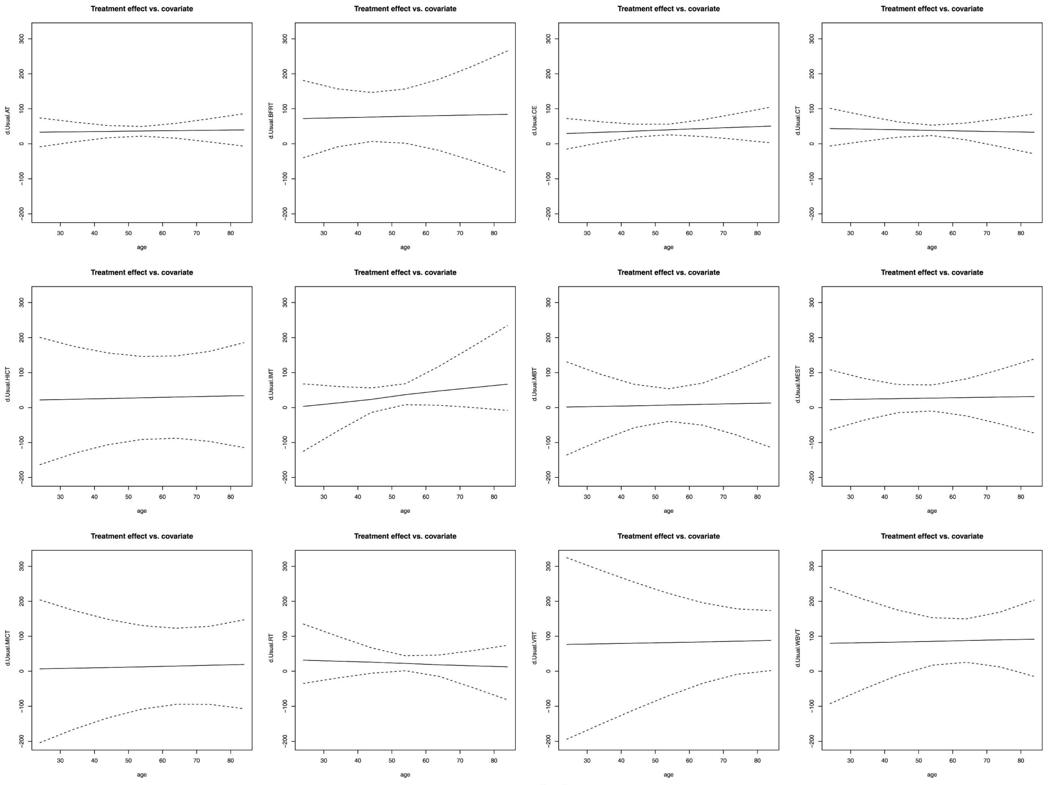

A(a)

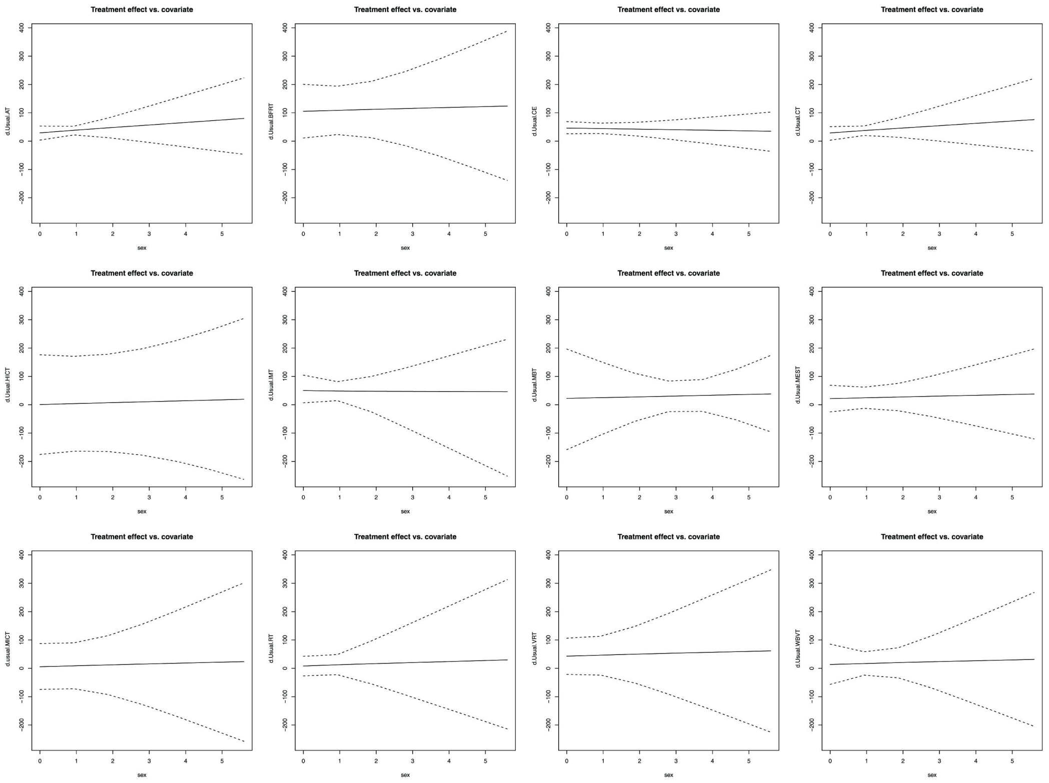

A(b)

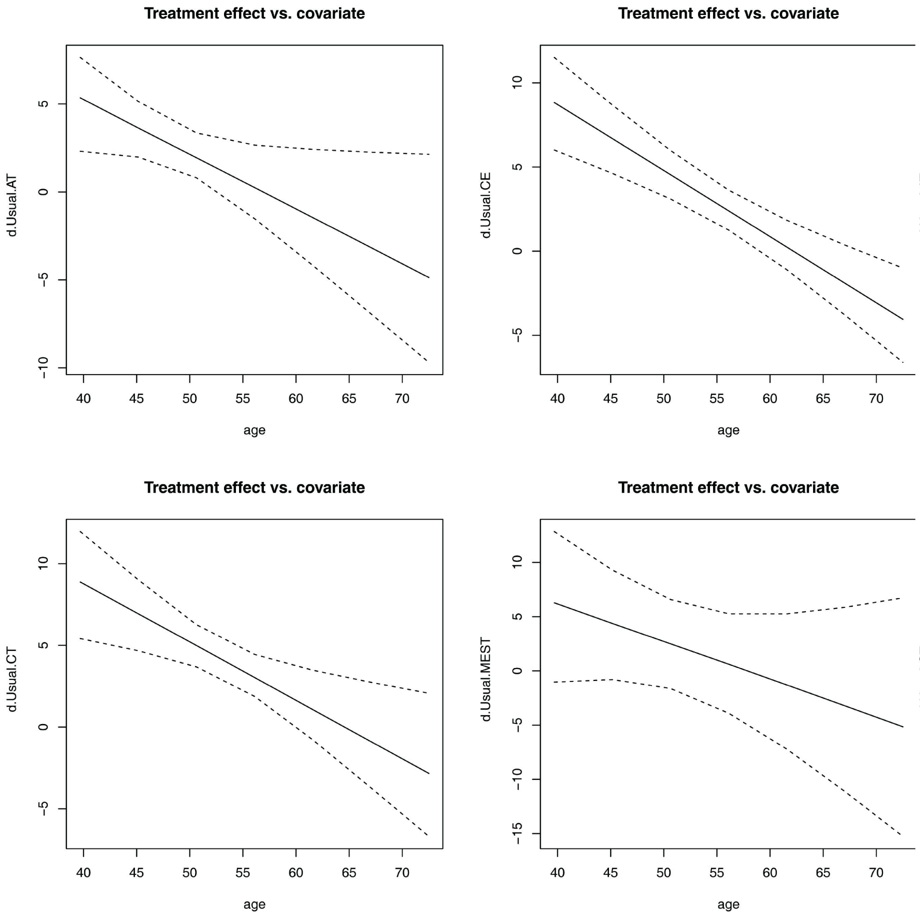
Treatment effect vs. covariate
d.Usual.AT
d.Usual.CE
d.Usual.CT
d.Usual.MEST
age

B(a)

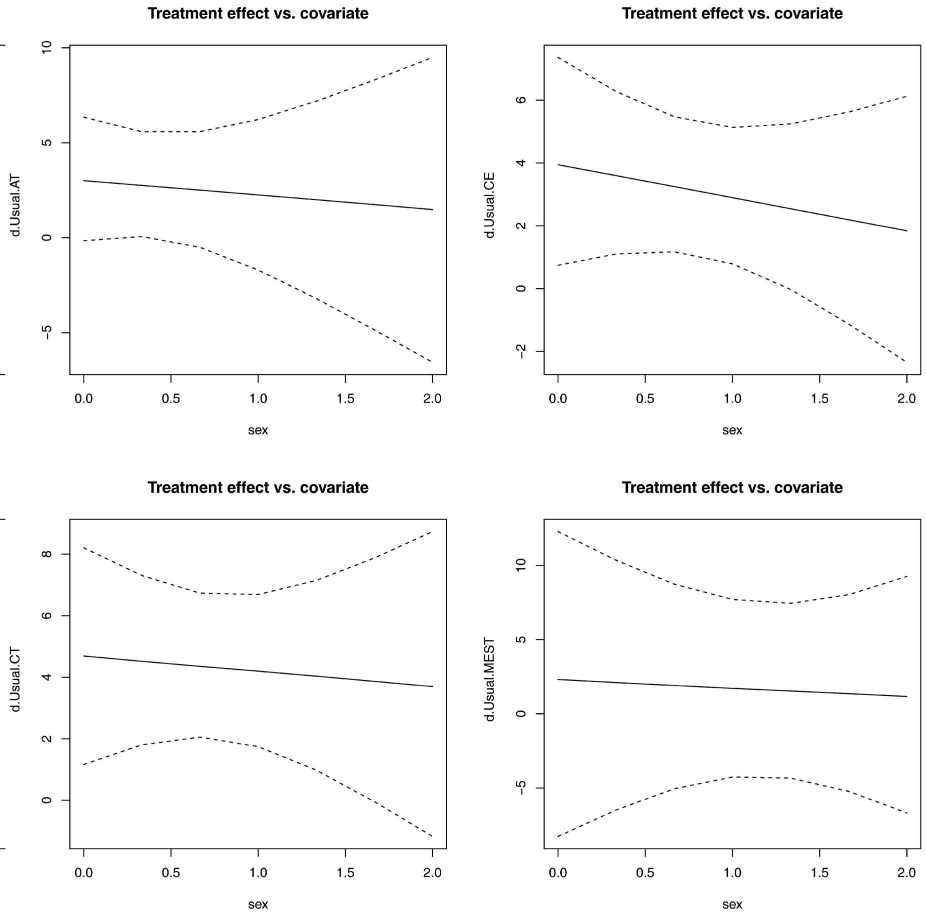
Treatment effect vs. covariate
d.Usual.AT
d.Usual.CE
d.Usual.CT
d.Usual.MEST
sex

B(b)

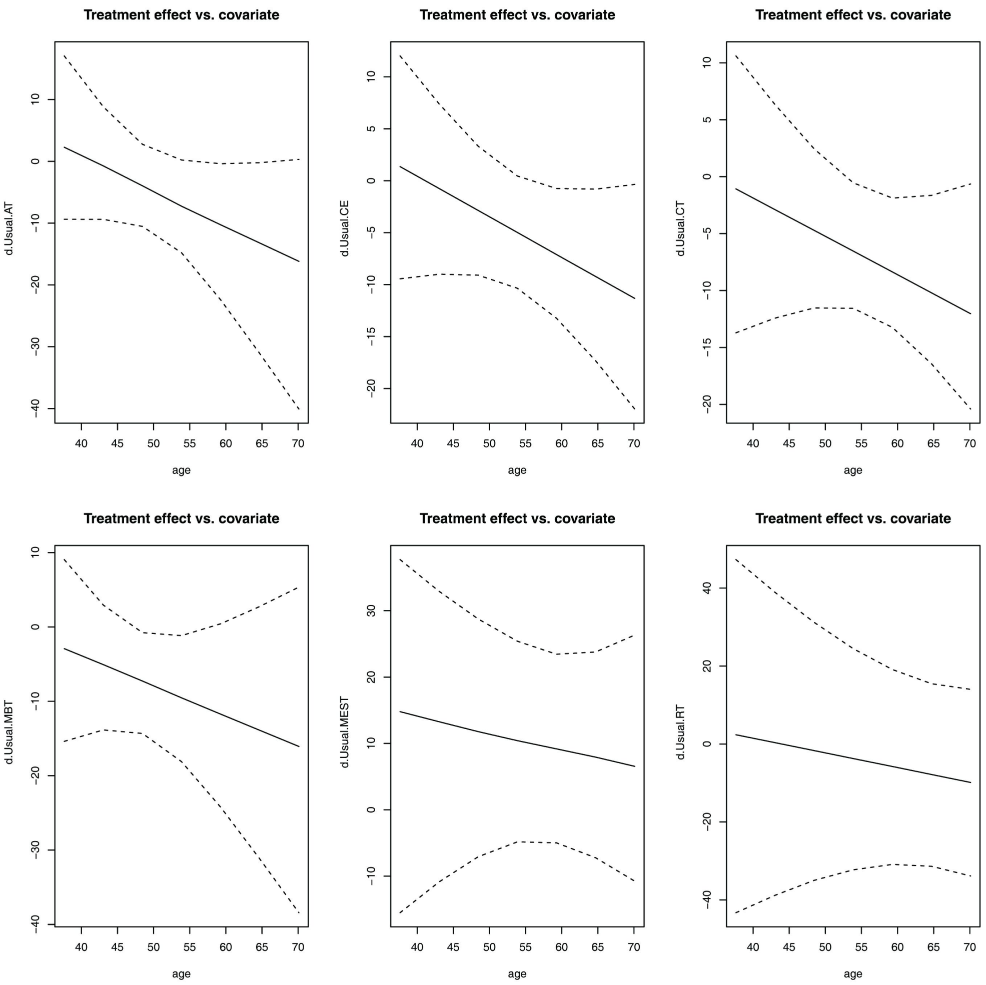
Treatment effect vs. covariate
d.Usual.AT
age
Treatment effect vs. covariate
d.Usual.CE
age
Treatment effect vs. covariate
d.Usual.CT
age
Treatment effect vs. covariate
d.Usual.MBT
age
Treatment effect vs. covariate
d.Usual.MEST
age
Treatment effect vs. covariate
d.Usual.RT
age

C(a)

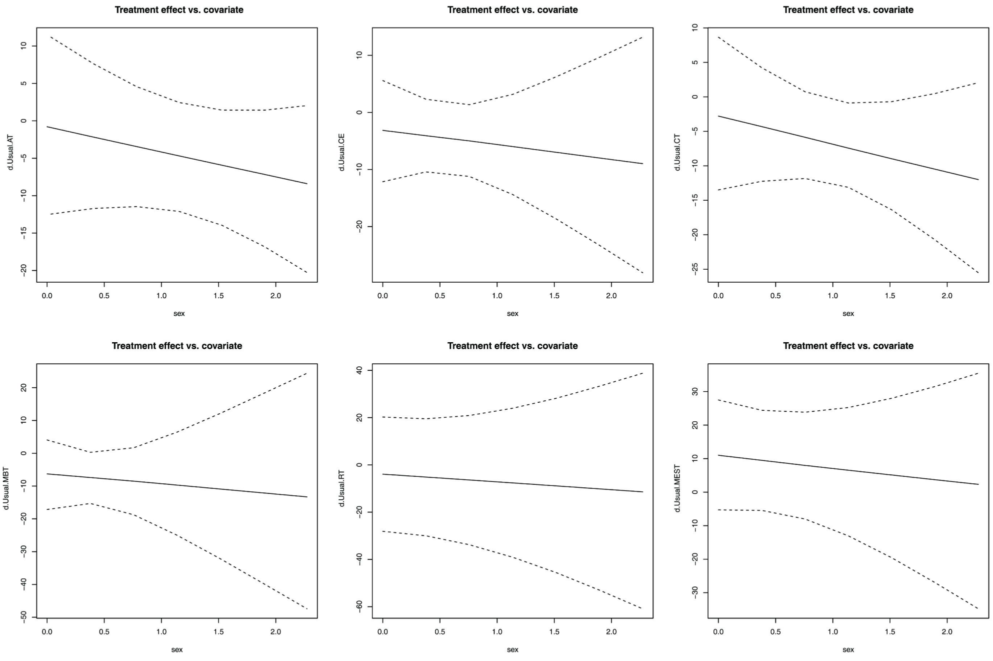
Treatment effect vs. covariate
d.Usual.AT
sex
Treatment effect vs. covariate
d.Usual.CE
sex
Treatment effect vs. covariate
d.Usual.CT
sex
Treatment effect vs. covariate
d.Usual.MBT
sex
Treatment effect vs. covariate
d.Usual.RT
sex
Treatment effect vs. covariate
d.Usual.MEST
sex

C(b)

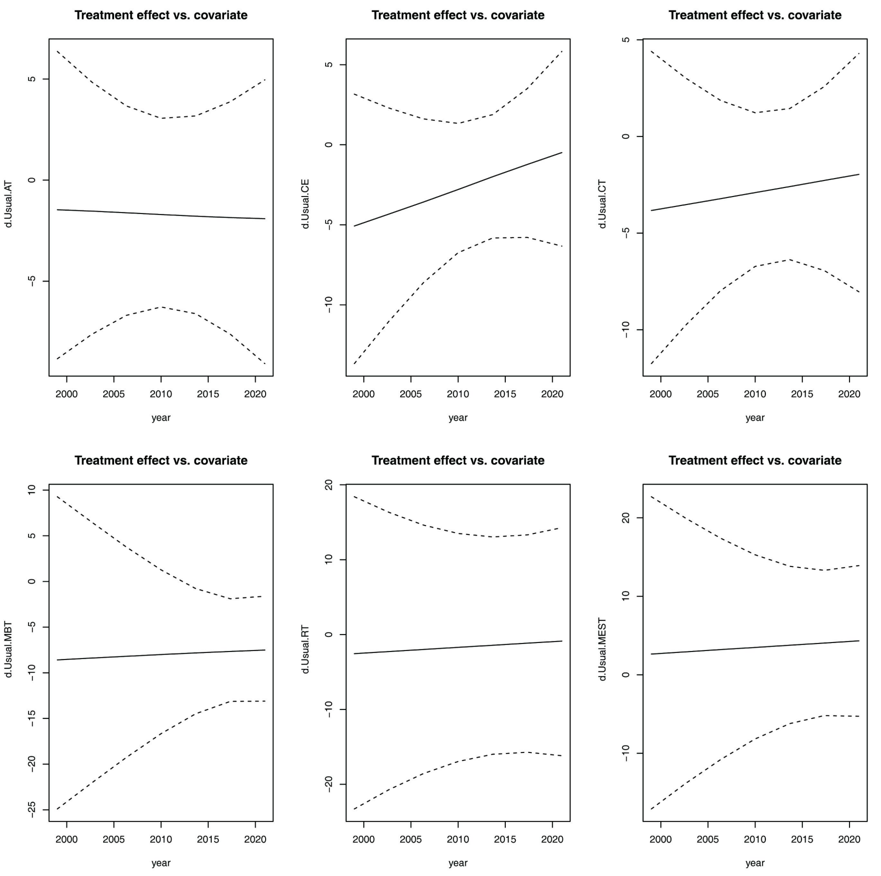
Treatment effect vs. covariate
d.Usual.AT
year
Treatment effect vs. covariate
d.Usual.CE
year
Treatment effect vs. covariate
d.Usual.CT
year
Treatment effect vs. covariate
d.Usual.MBT
year
Treatment effect vs. covariate
d.Usual.RT
year
Treatment effect vs. covariate
d.Usual.MEST
year

D(a)

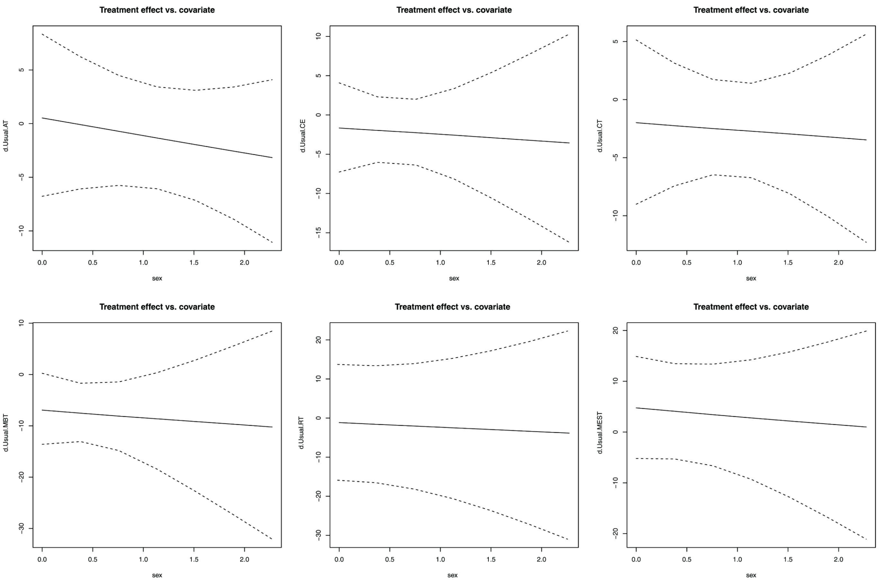
Treatment effect vs. covariate
d.Usual.AT
sex
Treatment effect vs. covariate
d.Usual.CE
sex
Treatment effect vs. covariate
d.Usual.CT
sex
Treatment effect vs. covariate
d.Usual.MBT
sex
Treatment effect vs. covariate
d.Usual.RT
sex
Treatment effect vs. covariate
d.Usual.MEST
sex

D(b)

Network Meta-regression analysis for the four outcomes with covariates (age and sex ratio).
A: Six-Minute Walkability.
B: Peak oxygen uptake.
C: systolic blood pressure.
D: diastolic blood pressure.
a: age. b: sex ratio.
The abscissa is the value of the covariate and the ordinate is the relative efficacy of the interventions. Curves are presented as mean and confidence interval of 95%.
AT=aerobic training. Usual=Usual care. CT=combined training. RT= resistance training. CE= Cycle Dynamometers. MEST=Electrical muscle stimulation. IMT=Inspiratory muscle training. BFRT=Blood flow restriction exercise. HICT=High-intensity circuit training. MICT=Medium intensity continuous training. VRT=Virtual Reality Training. MBT=Body and mind training.

Appendix K. Confidence in network meta analysis (CINeMA)

Table. CINeMA tool for the results of the effect of different exercise modalities on 6-minute walking capacity in hemodialysis patients.

	Quality assessment						Confidence rating	Reason(s)for downgrading
	Within-study bias	Reporting bias	Indirectness	Imprecision	Heterogeneity	Incoherence		
AT vs CE	Some concerns	Low risk	No concerns	Major concerns	No concerns	No concerns	Very low	①④
AT vs Usual	Some concerns	Low risk	No concerns	No concerns	Major concerns	No concerns	Very low	①②
AT vs RT	Some concerns	Low risk	No concerns	Major concerns	No concerns	No concerns	Very low	①④
BFRT vs CE	Some concerns	Low risk	No concerns	Major concerns	No concerns	No concerns	Very low	①④
BFRT vs Usual	Some concerns	Low risk	No concerns	No concerns	No concerns	No concerns	Moderate	①
CE vs Usual	Some concerns	Low risk	No concerns	No concerns	No concerns	No concerns	Moderate	①
CE vs MEST	Some concerns	Low risk	No concerns	Major concerns	No concerns	No concerns	Very low	①④
Usual vs CT	Some concerns	Low risk	No concerns	No concerns	No concerns	No concerns	Moderate	①
CT vs VRT	Some concerns	Low risk	No concerns	Major concerns	No concerns	No concerns	Very low	①④
Usual vs HICT	Some concerns	Low risk	No concerns	Major concerns	No concerns	No concerns	Very low	①④
Usual vs IMT	Some concerns	Low risk	No concerns	No concerns	Major concerns	No concerns	Very low	①②
Usual vs MBT	Major concerns	Low risk	No concerns	Major concerns	No concerns	No concerns	Very low	①④

Usual vs MEST	Some concerns	Low risk	No concerns	Major concerns	No concerns	No concerns	Very low	①④
Usual vs MICT	Some concerns	Low risk	No concerns	Major concerns	No concerns	No concerns	Very low	①④
Usual vs RT	Some concerns	Low risk	No concerns	No concerns	Major concerns	No concerns	Very low	①②
Usual vs WBVT	Some concerns	Low risk	No concerns	Major concerns	No concerns	No concerns	Very low	①④
HICT vs MICT	Some concerns	Low risk	No concerns	Major concerns	No concerns	No concerns	Very low	①④
IMT vs RT	Some concerns	Low risk	No concerns	Major concerns	No concerns	No concerns	Very low	①④
AT vs BFRT	Some concerns	Low risk	No concerns	Major concerns	No concerns	No concerns	Very low	①④
AT vs CT	Some concerns	Low risk	No concerns	Major concerns	No concerns	No concerns	Very low	①④
AT vs HICT	Some concerns	Low risk	No concerns	Major concerns	No concerns	No concerns	Very low	①④
AT vs IMT	Some concerns	Low risk	No concerns	Major concerns	No concerns	No concerns	Very low	①④
AT vs MBT	Major concerns	Low risk	No concerns	Major concerns	No concerns	No concerns	Very low	①④
AT vs MEST	Some concerns	Low risk	No concerns	Major concerns	No concerns	No concerns	Very low	①
AT vs MICT	Some concerns	Low risk	No concerns	Major concerns	No concerns	No concerns	Very low	①
AT vs VRT	Some concerns	Low risk	No concerns	Major concerns	No concerns	No concerns	Very low	①

AT vs WBVT	Some concerns	Low risk	No concerns	Major concerns	No concerns	No concerns	Very low	①
BFRT vs CT	Some concerns	Low risk	No concerns	Major concerns	No concerns	No concerns	Very low	①④
BFRT vs HICT	Some concerns	Low risk	No concerns	Major concerns	No concerns	No concerns	Very low	①④
BFRT vs IMT	Some concerns	Low risk	No concerns	Major concerns	No concerns	No concerns	Very low	①④
BFRT vs MBT	Some concerns	Low risk	No concerns	Major concerns	No concerns	No concerns	Very low	①④
BFRT vs MEST	Some concerns	Low risk	No concerns	Major concerns	No concerns	No concerns	Very low	①④
BFRT vs MICT	Some concerns	Low risk	No concerns	Major concerns	No concerns	No concerns	Very low	①④
BFRT vs RT	Some concerns	Low risk	No concerns	Major concerns	No concerns	No concerns	Very low	①④
BFRT vs VRT	Some concerns	Low risk	No concerns	Major concerns	No concerns	No concerns	Very low	①④
BFRT vs WBVT	Some concerns	Low risk	No concerns	Major concerns	No concerns	No concerns	Very low	①④
CE vs CT	Some concerns	Low risk	No concerns	Major concerns	No concerns	No concerns	Very low	①④
CE vs HICT	Some concerns	Low risk	No concerns	Major concerns	No concerns	No concerns	Very low	①④
CE vs IMT	Some concerns	Low risk	No concerns	Major concerns	No concerns	No concerns	Very low	①④
CE vs MBT	Major concerns	Low risk	No concerns	Major concerns	No concerns	No concerns	Very low	①④

CE vs MICT	Some concerns	Low risk	No concerns	Major concerns	No concerns	No concerns	Very low	①④
CE vs RT	Some concerns	Low risk	No concerns	Major concerns	No concerns	No concerns	Very low	①④
CE vs VRT	Some concerns	Low risk	No concerns	Major concerns	No concerns	No concerns	Very low	①④
CE vs WBVT	Some concerns	Low risk	No concerns	Major concerns	No concerns	No concerns	Very low	①④
CT vs HICT	Some concerns	Low risk	No concerns	Major concerns	No concerns	No concerns	Very low	①④
CT vs IMT	Some concerns	Low risk	No concerns	Major concerns	No concerns	No concerns	Very low	①④
CT vs MBT	Some concerns	Low risk	No concerns	Major concerns	No concerns	No concerns	Very low	①④
CT vs MEST	Some concerns	Low risk	No concerns	Major concerns	No concerns	No concerns	Very low	①④
CT vs MICT	Some concerns	Low risk	No concerns	Major concerns	No concerns	No concerns	Very low	①④
CT vs RT	Some concerns	Low risk	No concerns	Major concerns	No concerns	No concerns	Very low	①④
CT vs WBVT	Some concerns	Low risk	No concerns	Major concerns	No concerns	No concerns	Very low	①④
Usual vs VRT	Some concerns	Low risk	No concerns	Major concerns	No concerns	No concerns	Very low	①④
HICT vs IMT	Some concerns	Low risk	No concerns	Major concerns	No concerns	No concerns	Very low	①④
HICT vs MBT	Some concerns	Low risk	No concerns	Major concerns	No concerns	No concerns	Very low	①④

HICT vs MEST	Some concerns	Low risk	No concerns	Major concerns	No concerns	No concerns	Very low	①④
HICT vs RT	Some concerns	Low risk	No concerns	Major concerns	No concerns	No concerns	Very low	①④
HICT vs VRT	Some concerns	Low risk	No concerns	Major concerns	No concerns	No concerns	Very low	①④
HICT vs WBVT	Some concerns	Low risk	No concerns	Major concerns	No concerns	No concerns	Very low	①④
IMT vs MBT	Major concerns	Low risk	No concerns	Major concerns	No concerns	No concerns	Very low	①④
IMT vs MEST	Some concerns	Low risk	No concerns	Major concerns	No concerns	No concerns	Very low	①④
IMT vs MICT	Some concerns	Low risk	No concerns	Major concerns	No concerns	No concerns	Very low	①④
IMT vs VRT	Some concerns	Low risk	No concerns	Major concerns	No concerns	No concerns	Very low	①④
IMT vs WBVT	Some concerns	Low risk	No concerns	Major concerns	No concerns	No concerns	Very low	①④
MBT vs MEST	Major concerns	Low risk	No concerns	Major concerns	No concerns	No concerns	Very low	④
MBT vs MICT	Some concerns	Low risk	No concerns	Major concerns	No concerns	No concerns	Very low	①④
MBT vs RT	Some concerns	Low risk	No concerns	Major concerns	No concerns	No concerns	Very low	①④
MBT vs VRT	Some concerns	Low risk	No concerns	Major concerns	No concerns	No concerns	Very low	①④
MBT vs WBVT	Major concerns	Low risk	No concerns	Major concerns	No concerns	No concerns	Very low	①④

MEST vs MICT	Some concerns	Low risk	No concerns	Major concerns	No concerns	No concerns	Very low	①④
MEST vs RT	Some concerns	Low risk	No concerns	Major concerns	No concerns	No concerns	Very low	①④
MEST vs VRT	Some concerns	Low risk	No concerns	Major concerns	No concerns	No concerns	Very low	①④
MEST vs WBVT	Some concerns	Low risk	No concerns	Major concerns	No concerns	No concerns	Very low	①④
MICT vs RT	Some concerns	Low risk	No concerns	Major concerns	No concerns	No concerns	Very low	①④
MICT vs VRT	Some concerns	Low risk	No concerns	Major concerns	No concerns	No concerns	Very low	①④
MICT vs WBVT	Some concerns	Low risk	No concerns	Major concerns	No concerns	No concerns	Very low	①④
RT vs VRT	Some concerns	Low risk	No concerns	Major concerns	No concerns	No concerns	Very low	①④
RT vs WBVT	Some concerns	Low risk	No concerns	Major concerns	No concerns	No concerns	Very low	①④
VRT vs WBVT	Some concerns	Low risk	No concerns	Major concerns	No concerns	No concerns	Very low	①④

Table. CINeMA tool for the results of the effect of different exercise modalities on peak oxygen uptake in hemodialysis patients.

	Quality assessment						Confidence rating	Reason(s) for downgrading
	Within-study bias	Reporting bias	Indirectness	Imprecision	Heterogeneity	Incoherence		
AT vs CE	Some concerns	Low risk	No concerns	No concerns	Major concerns	Major concerns	Very low	①②③
AT vs Usual	Some concerns	Low risk	No concerns	No concerns	No concerns	Major concerns	Very low	①③
CE vs CT	Some concerns	Low risk	No concerns	Major concerns	No concerns	No concerns	Very low	①④
CE vs Usual	Some concerns	Low risk	No concerns	No concerns	No concerns	No concerns	Moderate	①
CE vs MEST	Some concerns	Low risk	No concerns	Major concerns	No concerns	No concerns	Very low	①④
CE vs MICT	Some concerns	Low risk	No concerns	Major concerns	No concerns	No concerns	Very low	①④
Usual vs CT	Some concerns	Low risk	No concerns	No concerns	No concerns	No concerns	Moderate	①
CT vs MICT	Some concerns	Low risk	No concerns	Major concerns	No concerns	No concerns	Very low	①④
Usual vs HICT	Some concerns	Low risk	No concerns	Major concerns	No concerns	Major concerns	Very low	①③④
Usual vs MEST	Some concerns	Low risk	No concerns	Major concerns	No concerns	No concerns	Very low	①④
Usual vs MICT	Some concerns	Low risk	No concerns	No concerns	No concerns	No concerns	Moderate	①
AT vs CT	Some concerns	Low risk	No concerns	Major concerns	No concerns	Major concerns	Very low	①③④

AT vs HICT	Some concerns	Low risk	No concerns	Major concerns	No concerns	Major concerns	Very low	①③④
AT vs MEST	Some concerns	Low risk	No concerns	Major concerns	No concerns	Major concerns	Very low	①③④
AT vs MICT	Some concerns	Low risk	No concerns	Major concerns	No concerns	Major concerns	Very low	①③④
CE vs HICT	Some concerns	Low risk	No concerns	Major concerns	No concerns	Major concerns	Very low	①③④
CT vs HICT	Some concerns	Low risk	No concerns	Major concerns	No concerns	Major concerns	Very low	①③④
CT vs MEST	Some concerns	Low risk	No concerns	Major concerns	No concerns	Major concerns	Very low	①③④
HICT vs MEST	Some concerns	Low risk	No concerns	Major concerns	No concerns	Major concerns	Very low	①③④
HICT vs MICT	Some concerns	Low risk	No concerns	Major concerns	No concerns	Major concerns	Very low	①③④
MEST vs MICT	Some concerns	Low risk	No concerns	Major concerns	No concerns	Major concerns	Very low	①③④

Table. CINeMA tool for the results of the effect of different exercise modalities on systolic blood pressure in hemodialysis patients.

	Quality assessment						Confidence rating	Reason(s) for downgrading
	Within-study bias	Reporting bias	Indirectness	Imprecision	Heterogeneity	Incoherence		
AT vs CE	Some concerns	Low risk	No concerns	Major concerns	No concerns	No concerns	Very low	①④
AT vs CT	Some concerns	Low risk	No concerns	Major concerns	No concerns	No concerns	Very low	①④
AT vs Usual	Some concerns	Low risk	No concerns	Major concerns	No concerns	No concerns	Very low	①④
CE vs CT	Some concerns	Low risk	No concerns	Major concerns	No concerns	No concerns	Very low	①④
CE vs Usual	Some concerns	Low risk	No concerns	Major concerns	No concerns	No concerns	Very low	①④
CE vs MEST	Some concerns	Low risk	No concerns	Major concerns	No concerns	No concerns	Very low	①④
CE vs RT	No concerns	Low risk	No concerns	Major concerns	No concerns	No concerns	Low	④
Usual vs CT	Some concerns	Low risk	No concerns	No concerns	Major concerns	No concerns	Very low	①②
CT vs RT	No concerns	Low risk	No concerns	Major concerns	No concerns	No concerns	Low	④
Usual vs MBT	Some concerns	Low risk	No concerns	No concerns	Major concerns	No concerns	Very low	①②
Usual vs MEST	Some concerns	Low risk	No concerns	Major concerns	No concerns	No concerns	Very low	①④
Usual vs RT	No concerns	Low risk	No concerns	Major concerns	No concerns	No concerns	Low	④

AT vs MBT	Some concerns	Low risk	No concerns	Major concerns	No concerns	No concerns	Very low	①④
AT vs MEST	Some concerns	Low risk	No concerns	Major concerns	No concerns	No concerns	Very low	①④
AT vs RT	Some concerns	Low risk	No concerns	Major concerns	No concerns	No concerns	Very low	①④
CE vs MBT	Some concerns	Low risk	No concerns	Major concerns	No concerns	No concerns	Very low	①④
CT vs MBT	Some concerns	Low risk	No concerns	Major concerns	No concerns	No concerns	Very low	①④
CT vs MEST	Some concerns	Low risk	No concerns	No concerns	Major concerns	No concerns	Very low	①②
MBT vs MEST	Some concerns	Low risk	No concerns	No concerns	Major concerns	No concerns	Very low	①②
MBT vs RT	Some concerns	Low risk	No concerns	Major concerns	No concerns	No concerns	Very low	①④
MEST vs RT	Some concerns	Low risk	No concerns	Major concerns	No concerns	No concerns	Very low	①④

Table. CINeMA tool for the results of the effect of different exercise modalities on diastolic blood pressure in hemodialysis patients.

	Quality assessment						Confidence rating	Reason(s) for downgrading
	Within-study bias	Reporting bias	Indirectness	Imprecision	Heterogeneity	Incoherence		
AT vs CE	Some concerns	Low risk	No concerns	Major concerns	No concerns	No concerns	Very low	①④
AT vs CT	Some concerns	Low risk	No concerns	Major concerns	No concerns	No concerns	Very low	①④
AT vs Usual	Some concerns	Low risk	No concerns	Major concerns	No concerns	No concerns	Very low	①④
CE vs CT	Some concerns	Low risk	No concerns	Major concerns	No concerns	No concerns	Very low	①④
CE vs Usual	Some concerns	Low risk	No concerns	Major concerns	No concerns	No concerns	Very low	①④
CE vs MEST	Some concerns	Low risk	No concerns	Major concerns	No concerns	No concerns	Very low	①④
CE vs RT	No concerns	Low risk	No concerns	Major concerns	No concerns	No concerns	Low	④
Usual vs CT	Some concerns	Low risk	No concerns	No concerns	Major concerns	No concerns	Very low	①②
CT vs RT	No concerns	Low risk	No concerns	Major concerns	No concerns	No concerns	Low	④
Usual vs MBT	Some concerns	Low risk	No concerns	No concerns	No concerns	No concerns	Moderate	①
Usual vs MEST	Some concerns	Low risk	No concerns	Major concerns	No concerns	No concerns	Very low	①④

Usual vs RT	No concerns	Low risk	No concerns	Major concerns	No concerns	No concerns	Low	④
AT vs MBT	Some concerns	Low risk	No concerns	No concerns	Major concerns	No concerns	Very low	①②
AT vs MEST	Some concerns	Low risk	No concerns	Major concerns	No concerns	No concerns	Very low	①④
AT vs RT	Some concerns	Low risk	No concerns	Major concerns	No concerns	No concerns	Very low	①④
CE vs MBT	Some concerns	Low risk	No concerns	No concerns	Major concerns	No concerns	Very low	①②
CT vs MBT	Some concerns	Low risk	No concerns	Major concerns	No concerns	No concerns	Very low	①④
CT vs MEST	Some concerns	Low risk	No concerns	Major concerns	No concerns	No concerns	Very low	①④
MBT vs MEST	Some concerns	Low risk	No concerns	No concerns	No concerns	No concerns	Moderate	①
MBT vs RT	Some concerns	Low risk	No concerns	Major concerns	No concerns	No concerns	Very low	①④
MEST vs RT	Some concerns	Low risk	No concerns	Major concerns	No concerns	No concerns	Very low	①④

①: within-study bias

②: heterogeneity

③: incoherence

④: imprecision

AppendixL. Publication bias

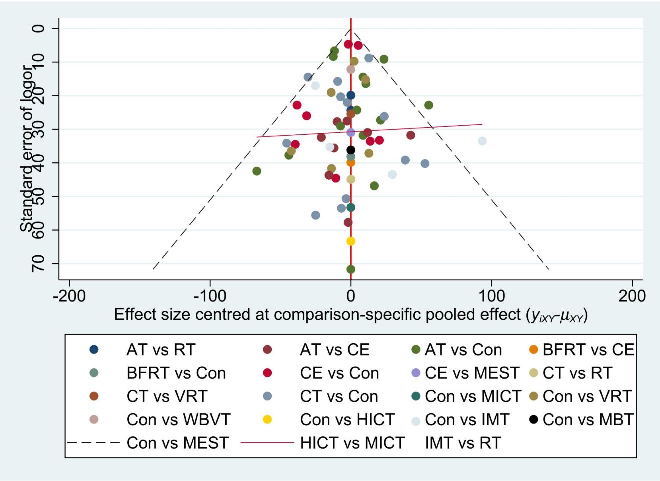

(a)

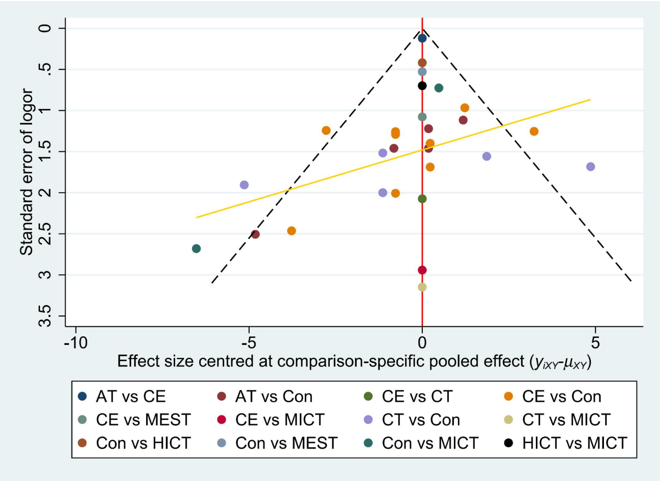

(b)

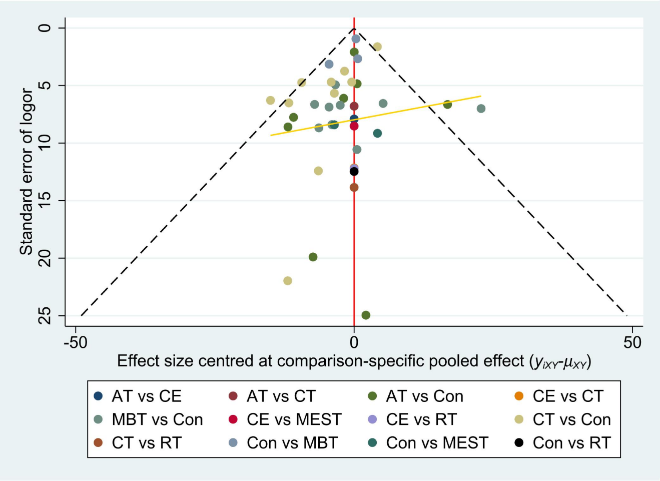

(c)

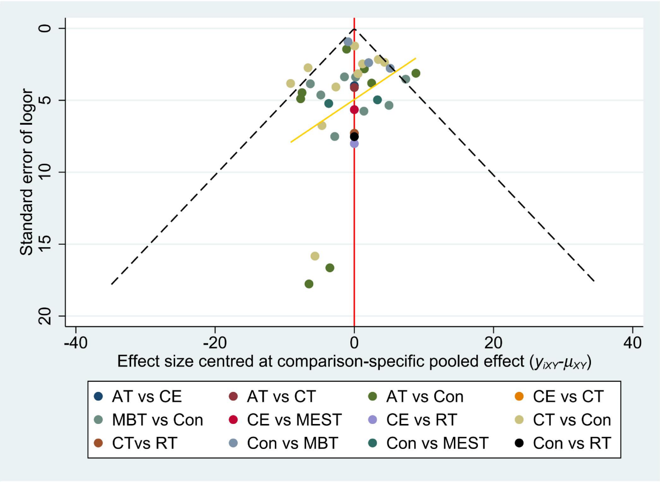

(d)

Network meta-analysis funnel plots for the assessment of publication bias in studies in patients with hemodialysis. Different points represent comparisons of different interventions.AT=aerobic training. Con=Usual care. CT=combined training. RT= resistance training. CE= Cycle Dynamometers. MEST=Electrical muscle stimulation. IMT=Inspiratory muscle training. BFRT=Blood flow restriction exercise. HICT=High-intensity circuit training. MICT=Medium intensity continuous training. WBVT=Whole Body Vibration Training. VRT=Virtual Reality Training. MBT=Body and mind training.
